# Detailed analysis of Mdivi-1 effects on complex I and respiratory supercomplex assembly

**DOI:** 10.1038/s41598-024-69748-y

**Published:** 2024-08-24

**Authors:** Nico Marx, Nadine Ritter, Paul Disse, Guiscard Seebohm, Karin B. Busch

**Affiliations:** 1 Department of Biology, Institute of Integrative Cell Biology and Physiology (IIZP), University of Münster, Schloßplatz 5, 48149 Münster, Germany; 2 Department of Cardiovascular Medicine, Institute for Genetics of Heart Diseases (IfGH), University Hospital Münster, 48149 Münster, Germany; 3 Department of Drug Design and Pharmacology, University of Copenhagen, 2100 Copenhagen, Denmark

**Keywords:** Mitochondria, Respiratory complex I, Mdivi-1, Inhibition, Respiratory supercomplexes, Neuronal activity, Calcium metabolism, Cell biology, Cellular imaging, Organelles

## Abstract

Several human diseases, including cancer and neurodegeneration, are associated with excessive mitochondrial fragmentation. In this context, mitochondrial division inhibitor (Mdivi-1) has been tested as a therapeutic to block the fission-related protein dynamin-like protein-1 (Drp1). Recent studies suggest that Mdivi-1 interferes with mitochondrial bioenergetics and complex I function. Here we show that the molecular mechanism of Mdivi-1 is based on inhibition of complex I at the IQ site. This leads to the destabilization of complex I, impairs the assembly of N- and Q-respirasomes, and is associated with increased ROS production and reduced efficiency of ATP generation. Second, the calcium homeostasis of cells is impaired, which for example affects the electrical activity of neurons. Given the results presented here, a potential therapeutic application of Mdivi-1 is challenging because of its potential impact on synaptic activity. Similar to the Complex I inhibitor rotenone, Mdivi-1 may lead to neurodegenerative effects in the long term.

## Introduction

Neurons rely on mitochondrial ATP synthesis to fuel energy-intensive processes such as vesicle cycling and signal transduction. Consequently, mitochondria dysfunction is closely associated with neurodegenerative diseases^1^. In particular, respiratory Complex I (CI) inhibition seems to facilitate initiation of neurodegeneration^2,3^. Mammalian Complex I (CI), the NADH-ubiquinone oxidoreductase, is one of the largest multiprotein membrane complexes with a molecular weight of about 1 MDa. It consists of 45 subunits. CI has three catalytic functions: NADH oxidation, electron transfer to ubiquinone, and proton translocation. Together with respiratory complexes CIII and CIV, CI generates the proton motive force across the mitochondrial inner membrane (IMM), which drives ATP synthesis by ATP synthase (CV). CII is the succinate dehydrogenase, CIII the ubiquinol-cytochrome* c* oxidoreductase and CIV the cytochrome c-oxidase.

CI catalyzes the oxidation of NADH to NAD^+^ at the tip of the peripheral stalk. The electrons are transported via cofactors from the N-module to the Q-module. N- and Q-module build the hydrophilic peripheral arm. The flow of electrons alters the redox state of the protein and induces conformational changes of the protein. NADH-induced changes in the Q-cavity are only possible in the open conformation of the complex, while the reduction of quinone presumably happens only in the closed state^4^. The coupling mechanism between electron transfer and proton translocation in Complex I remains a controversial question in the field^5–7^. The membrane-embedded part, which transports protons from the matrix into the intra-cristae space, consists of the P_D_- and P_P_-module. Mutations in CI subunits can disrupt the proper assembly of CI, leading to unstable CI subassemblies and dysfunction including increased formation of reactive oxygen species (ROS)^8,9^. Chemical inhibition of CI can induce parkinsonism in humans. The inhibitor meperidine selectively targeted dopaminergic neurons of the *Substantia nigra* and induced dopamine deficiency and cell death^10^.

Complexes I, III and IV of the electron transport chain form larger supermolecular assemblies in different combinations, known as supercomplexes (SC)^11^. The N-respirasome contains I, III_2_ and IV_x_, while the Q-respirasome contains III_2_ and IV_x_. The plasticity model suggests a dynamic composition of single complexes and supercomplexes^12^.

The two assembly factors Cox7a2l (Supercomplex Assembly Factor I, SCAFI) and HIG1 Hypoxia Inducible Domain Family Member 2A (HIGD2A) are required for the assembly of supercomplexes^13,14^. SCAFI associates with complex III^15^ to recruit CIV for the formation of III + IV-containing supercomplexes^16^. In certain tissues, the COX7a2 isoform can replace SCAFI in the formation of Q-respirasomes. HIGD2A promotes the assembly of the COX3 module and associates with SCs to modulate the assembly of CIV in the SC^14^. Complex I itself appears to play a role in supercomplex assembly, but whether this requires a subcomplex^17^ or the full complex is not yet fully resolved. Vice versa, supercomplex formation appears to have a stabilizing effect on the individual complexes^18,19^. Several studies also indicate a functional advantage of SC formation, e.g. in increasing the efficiency of the electron transport chain^11,20,21^, or the minimization of mitochondrial ROS production^22,23^. Supercomplexes containing complexes I + III and IV of the respiratory chain are able to respire from any of their substrates^12^, resulting in higher metabolic flexibility and efficiency. Maintenance of CI and its supercomplexes is essential for mitochondrial structure and function^24^. Improper SC composition can lead to the formation of subcomplexes that impair mitochondrial function, as shown in a study with patient cells^17^.

In neurons, fusion and fission dynamics of mitochondria is important for the adaptation of mitochondrial and cellular functions^25^. So, mitochondrial fission is a prerequisite for the maturation of neuronal progenitor cells (NPCs) during neurogenesis^26,27^. Fission is mediated by the cytosolic dynamin-related protein Drp1, a GTPase. The chemical agent Mitochondrial Division Inhibitor-1 (Mdivi-1) is a commonly used inhibitor of Drp1 to suppress mitochondrial fission^28^, which is also effective in primary neurons. Several studies provided evidence of protective effects of fission-inhibition by Mdivi-1 in neurodegenerative models for Alzheimer’s disease (AD)^29,30^ and Parkinson’s disease (PD)^31,32^. However, more recent studies suggested that Mdivi-1 effects are more complex and target mitochondrial bioenergetics^33–35^. Clarification of this issue is important because inhibition of the ETC, in particular Complex I, is closely associated with onset of neurodegeneration^2,3^. Here we provide evidence that Mdivi-1 directly inhibits Complex I by binding to the I_Q_ site, deepening the observations made before by Bordt et al.^34^. This inhibition affects the assembly of Complex I and respiratory supercomplexes with consequences for ATP generation and mitochondrial calcium homeostasis. Ultimately, this leads to functional impairment of neurons.

## Results

### Mdivi-1 treatment causes decline of mitochondrial function and increases ROS levels

We first checked general Mdivi-1 effects on proliferation and morphology of cells. Long-term and acute treatment with Mdivi-1 significantly impaired the growth of HeLa cells (Supplementary Fig. S1). P21 expression, which is a cyclin-dependent kinase (CDK1) inhibitor, was increased and the morphology of neuronal progenitor cells (NPC) changed toward a round phenotype. In Hela cells, the mitochondrial mass per cell increased, as MitoTracker™Green (MTG) staining revealed. Also, the expression of the mitochondrial transcription factor A, TFAM, was increased indicating biogenesis of mitochondria. VDAC. As a second cell model, we chose human neurons that were differentiated from neuronal progenitor cells (NPC) (Supplementary Fig. S2). The generation of functional midbrain neurons was confirmed by gene-expression analysis and immune-staining of marker genes and the electric activity in response to different neurotransmitters was tested using a multi electrode array (MEA). The neuronal activity was primary responsive to glutamate/glycine and dopamine indicating a mixed neuronal cell culture.

To verify mitochondrial biogenesis, VDAC protein levels in three Mdivi-1 treated cell types (Hela, NPC and neurons) were determined. The VDAC protein levels, normalized on Tuj-1, a class III beta-tubulin, were significantly higher in Hela and NPC that were treated with Mdivi-1, while Neurons showed a tendency towards higher VDAC-protein levels (Supplementary Fig. S1). Together, this data indicates that Mdivi-1 affects cell proliferation, cellular shape and induces mitochondrial biogenesis. Mitochondrial biogenesis can be a stress response due to declining mitochondrial function.

**Figure 1:**
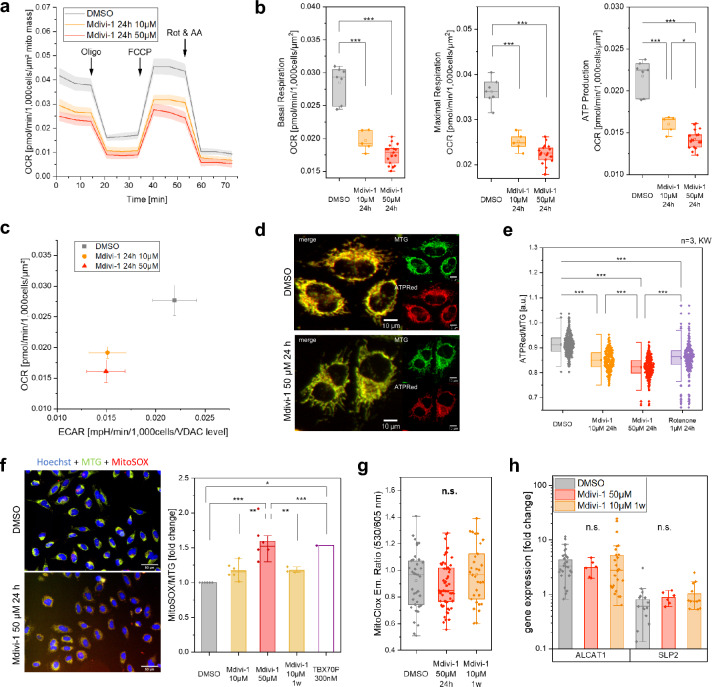
Mitochondrial function is reduced in Mdivi-1 treated cells. (**a**) Oxygen consumption rates (OCR) of control and Mdivi-1-treated HeLa cells, determined with an automatic flux analyzer (Seahorse XF96/Agilent) and normalized to mitochondrial area (µm^2^) per cell (mitochondrial area was determined by MiNA (Fig. S1)). Subsequent addition of an inhibitor for ATP synthase (oligomycin, 1.5 μM), uncoupler trifluoromethoxy carbonylcyanide phenylhydrazone (FCCP, 2 μM) and inhibitors for complex I (Rot., Rotenone; 0.5 μM) and complex III (AA; antimycin A; 1 μM). (**b**) Basal, maximal and ATP synthase linked respiration as oxygen consumption rate (OCR). (**c**) Metabolic profile changes as consequence of Mdivi-1 treatment, indicated as OCR/ECAR ratio, whereby ECAR is the extracellular acidification rate. (**d**,**e**) Mitochondrial ATP levels in Mdivi-1 treated and control HeLa cells as indicated by the fluorogenic dye ATPREd-1, co-staining with Mito Tracker Green (MTG) (N = 3, n_DMSO_ = 305, n_Mdivi-1/10 μM 24 h_ = 200, n_Mdivi-1 /50 μM 1w_ = 235, n_Rotenone 1 μM 24 h=_234). (**f**) Exemplary images of HeLa cells stained with Hoechst, MTG and MitoSOX for detection of superoxide. Superoxide levels of Mdivi-1 treated HeLa cells are elevated (N = 6, n≈10,800 per condition). (**g**) Determination of Cardiolipin peroxidation by MitoCLox staining in neurons treated with Mdivi-1 compared to DMSO. (N = 2, n_DMSO_ = 37, n_Mdivi-1 50 µM 24 h_ = 57, n_Mdivi-1 10 µM 1w_ = 31). (**h**) Gene expression of cardiolipin modifying proteins are not altered in Mdivi-1 treated cells compared to control neurons (N = 3, n = 9).

To test this, oxygen consumption rates (OCR) were measured with a real-time kinetic assay (Seahorse XF96 Analyzer/Agilent) (Fig. 1a). Basal, ATP synthesis-linked and maximal respiration were determined in control and Mdivi-1-treated cells before and after addition of the inhibitor oligomycin and the uncoupler FCCP, respectively. In the last step, complex I and complex III were inhibited by addition of rotenone and antimycin A (AA). OCR was normalized on the mitochondrial mass per cell. The basal, ATP synthesis-linked and maximal respiration in both, Hela cells (Fig. 1b) and neurons (Supplementary Fig. S3) were significantly decreased compared to the control. The OCR/ECAR ratio was significantly decreased in Mdivi-1-treated cells when normalized on the number of mitochondria per cell (Fig. 1c). This suggests that the observed biogenesis of mitochondria was as a stress response but could not fully compensate the decline of mitochondrial function. To further elucidate the consequences of decreased OCR, mitochondrial ATP levels were determined by using the fluorogenic dye ATPRed-1m, which fluorescence intensity is ATP-dependent (Fig. 1d). Indeed, relative mitochondrial ATP levels were decreased in Mdivi-1 treated cells. Remarkably, the effect was stronger than for the well characterized complex I inhibitor rotenone. Furthermore, Mdivi-1 treatment resulted in increased mitochondrial superoxide levels as determined by MitoSOX fluorescence (Fig. 1e,f). The lipid peroxidation sensor, MitoCLox^36^, did not indicate increased lipid peroxidation, though (Fig. 1g). Also, the expression of the cardiolipin modifying enzymes Acyl-CoA:lysocardiolipin acyltransferase-1 (ALCAT1) and Stomatin-like protein 2 (SLP2) were not significantly altered due to Mdivi-1 treatment (Fig. 1h). In sum, Mdivi-1 affected cell growth, shape, increased mitochondrial mass and compromised mitochondrial bioenergetics.

### Mdivi-1 reduces complex I assembly and affects supercomplex formation

**Figure 2:**
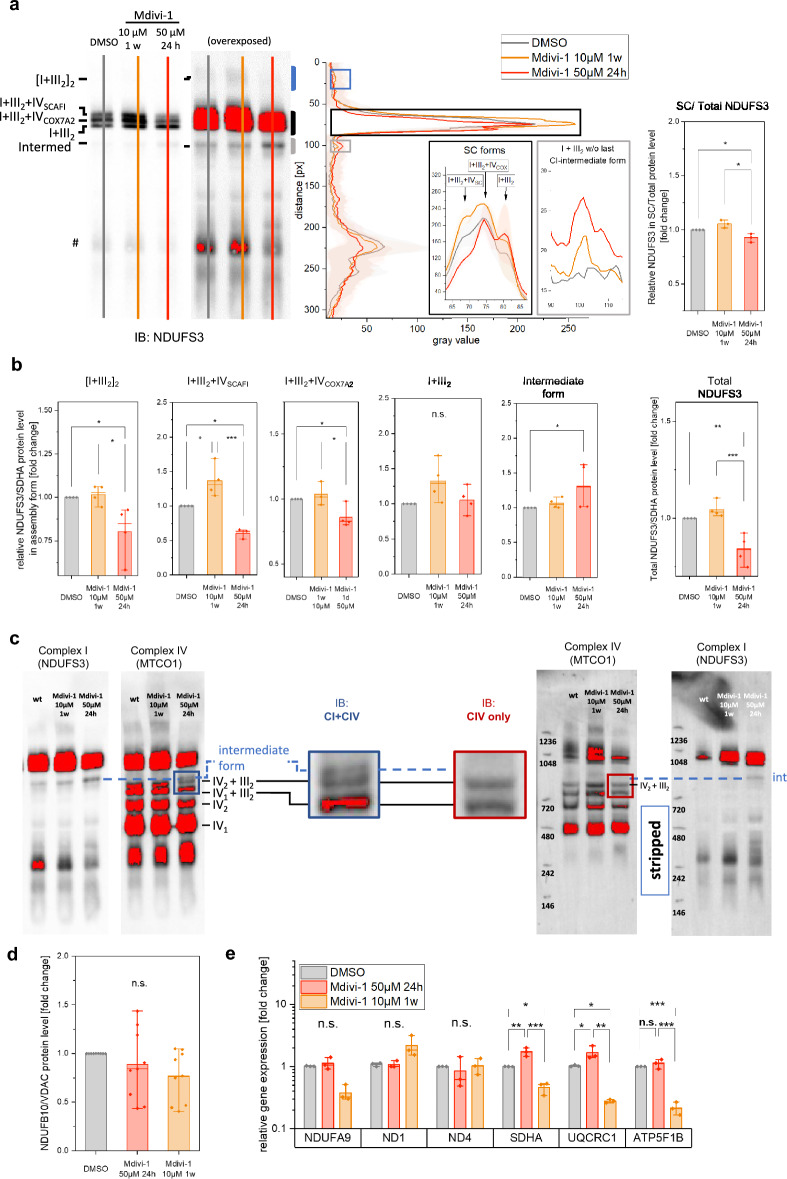
Mdivi-1 treatment impairs CI assembly into supercomplexes. (**a**) Blue-Native separation of proteins and immunoblotting. The line plot of Complex I subunit NDUFS3 shows differences in the assembly of CI and mitochondrial CI-supercomplexes (SC/total) containing CI in between Mdivi-1 treated and control HeLa cells (# = unspecific). (**b**) Quantification of CI assembly in different isoforms displays alterations between Mdivi-1 and DMSO treated HeLa cells (n = 4). I + III_2_ analyzed from blue frame region in (A). (**c**) Representative inverse immunoblotting with antibodies against NDUFS3 (complex I) and MTCO1 (complex IV). Overexposure visualizes that the intermediate complex I assembly form does not contain complex IV. (**d**) Protein level of the P-module subunit NDUFB10 is unaltered in Mdivi-1 treated HeLa cells. (N = 3, n = 9). (**e**) Gene expression of different OXPHOS subunits is altered due to Mdivi-1 treatment (N = 3 independent qPCRs, n = 4). Boxplots indicate median (line), 25th-75th percent percentile (box) and minimum and maximum values (whiskers). Statistics: one-way ANOVA. ***p ≤ 0.001; **p ≤ 0.01; **p ≤ 0.05.

To test, whether the assembly of OXPHOS complexes and supercomplexes (SC) was affected by Mdivi-1, we performed a Blue Native PAGE separation of the proteins. Quantification of the complex I (CI) core subunit NDUFS3 revealed that acute Mdivi-1 treatment reduced the amount of the N-respirasome I + III_2_ + IV (Fig. 2a). The SC I + III_2_ showed no significant change, but an intermediate NDUFS3 containing complex of 850 kDa was increased (Fig. 2a,b). The intermediate also contained the accessory subunit NDUFB10 (Supplementary Fig. S4). Immunoblotting of the same BN-PAGE with Anti-MTCO1 revealed that the intermediate form contained no complex IV (Fig. 2c). Thus, the intermediate SC form is rather a pre-CI or pre-CI + CIII_2_ than the pre-CI + CIII_2_ + CIV recently proposed^17^. Overexposing the immunoblot of the BN-PAGE revealed a CI-assembly with higher molecular weight. This is either the SC [I + III_2_]_2_, or the recently reported human megacomplex I_2_ + III_2_ + IV_2_^37^.

This SC was significantly decreased in acute Mdivi-1 treated HeLa cells compared to the control (DMSO treatment) (Fig. 2a, blue box in overexposed blot, and Fig. 2b). The detailed analysis showed reduced NDUFS3 protein levels in respirasomes I + III_2_ + IV_COX7A2_ as well as in I + III_2_ + IV_SCAFI_, which includes the supercomplex chaperone SCAFI in acute Mdivi-1 treated cells (Fig. 2b). Interestingly, acute treatment decreased this supercomplex form by 40%, while long-term treatment increased the level of I + III_2_ + IV_SCAFI_ by about 30%. It is intriguing to speculate that this specific increase in I + III_2_ + IV_SCAFI_ is linked to the observed increased ΔΨ_m_ in long-term treated cells. The total protein level of NDUFS3 in the BN-PAGE was decreased (Fig. 2B). Checking other complex I subunits showed that neither the NDUFB10 protein level, nor the expression levels of NDUFA9, ND1 and ND4 were altered (Fig. 2d,e). However, expression levels of the subunit SDHA of complex II and UQCRC1 of complex III were increased in acute Mdivi-1 treated HeLa cells while significantly decreased in long-term Mdivi-1 treated cells compared to the control (Fig. 2d). This suggests some adaptive dynamics in the make-up of the respiratory chain during ongoing Mdivi-1 treatment.

**Figure 3:**
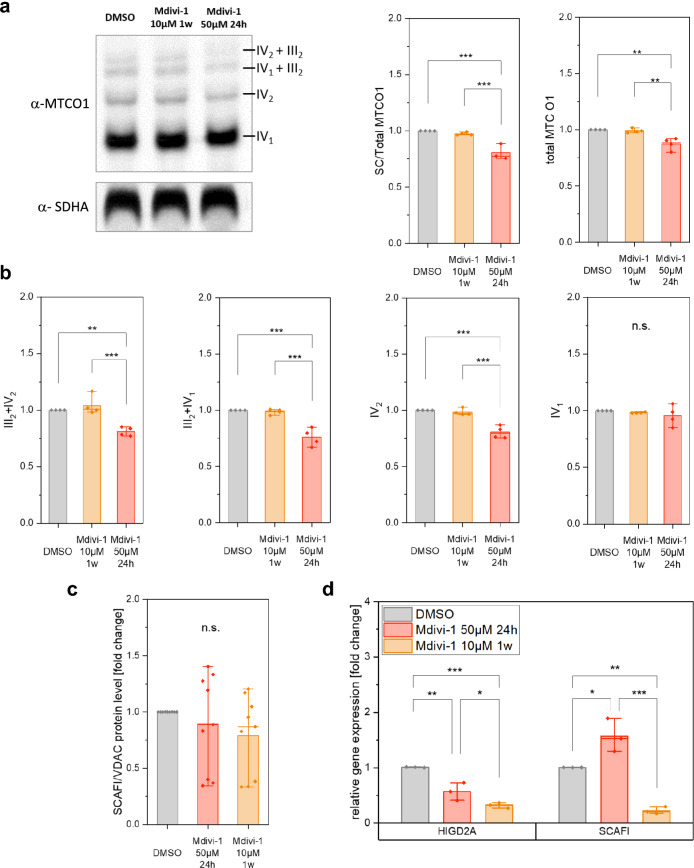
Acute Mdivi-1 treatment decreases the amount of Q-respirasomes. (**a**) Exemplary immunoblot of BN-PAGE with Mdivi-1 treated HeLa cells. Complex IV probed with α-MTCO1, Complex II probed with α-SDHA). (**b**) Quantification of supercomplexes reveals decreased supercomplex formation and dimerization of Complex IV (N = 4). (**c**) Expression of supercomplex promoting factors SCAFI and HIGD2A in Mdivi-1 treated cells (N = 3, n = 12). (**d**) Effects of Mdivi-1 treatment on protein levels of SCAFI and HIGD2A (N = 3, n = 9). Boxplots indicate median (line), 25th–75th percent percentile (box) and minimum and maximum values (whiskers). Statistics: one-way ANOVA with Tukey comparison. ***p ≤ 0.001; **p ≤ 0.01; **p ≤ 0.05.

To determine whether the compromised CI assembly affects the CIII + CIV supercomplexes, which comprise the Q-respirasome, their level was analyzed in the lower molecular weight region of the BN-PAGE. Indeed, the total amount of the supercomplexes III_2_ + IV_2_ and III_2_ + IV was decreased in HeLa cells by acute Mdivi-1 treatment (Fig. 3a,b). Also, the mean protein content of complex IV dimers was significantly reduced (− 20% ± 5% SD), while the amount of monomeric CIV was not altered. This reflects the significance of CI for the stability of CIV and CIV + CIII assembly.

The formation of supercomplexes is supported by assembly factors. We checked for changes in Supercomplex Assembly Factor I (SCAFI, also known as COX7A2L) and Hypoxia Inducible Domain Family Member 2A (HIGD2A). SCAFI promotes III/IV interaction^11,16^. On the protein level, total SCAFI protein as determined by WESTERN was not significantly changed in Mdivi-1 treated cells (Fig. 3c, and source data). This does not, however, indicate whether more or less SCAFI is bound to respiratory complexes. The expression of SCAFI in HeLa cells as determined by q-RT-PCR was significantly increased by acute Mdivi-1 treatment and decreased by long-term treatment, while the expression of HIGD2A was significantly decreased in acute and long-term Mdivi-1 treated cells. (Fig. 3d). Taken together, this data shows that acute Mdivi-1 treatment (50 µM, 24 h) impairs the formation of supercomplexes, resulting in the reduction of N- and Q-respirasomes. Long-term treatment (1 w) increases CI + CIII_2_ + CIV_SCAFI._

### Mdivi-1 inhibits complex I by blocking the quinone binding cavity

**Figure 4:**
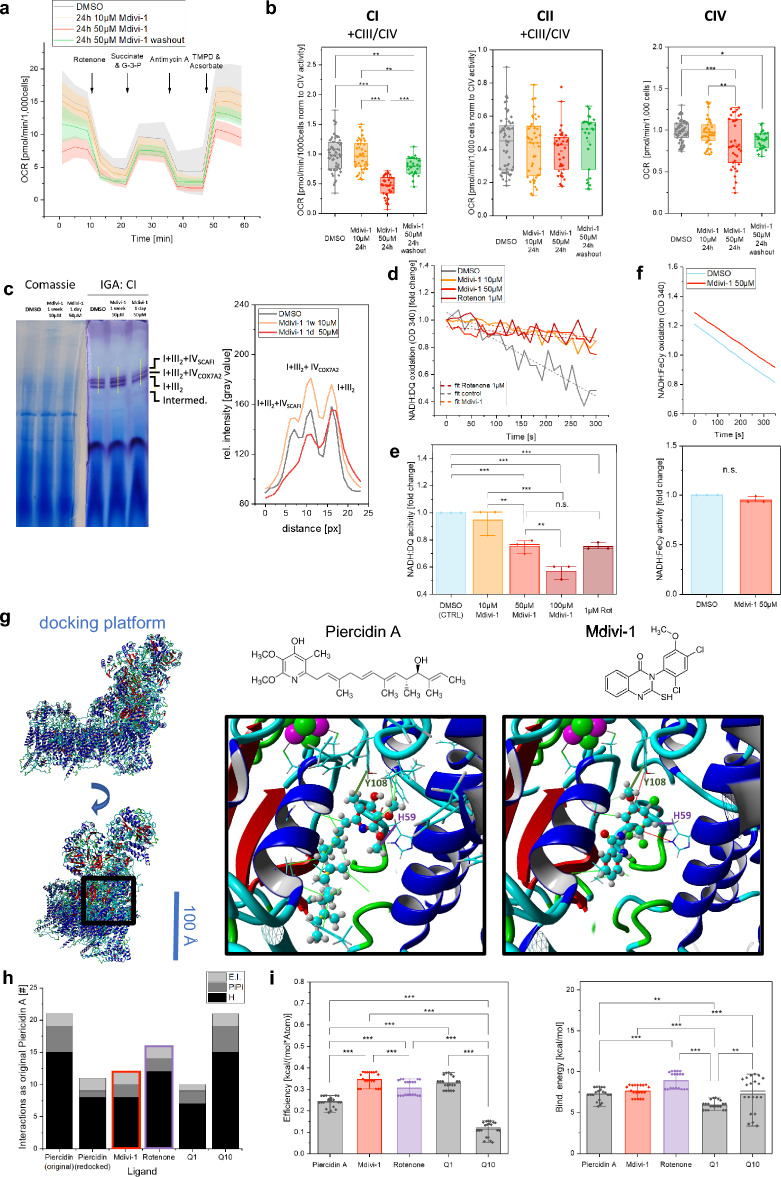
Inhibition of complex I by Mdivi-1 reduces supercomplex formation. (**a**,**b**) Determination of respiratory complex activities in HeLa cells by oxygen consumption rates (N = 3, n_DMSO_ = 41, n_Mdivi-1,10 µM, 1 w_ = 41, n_Mdivi-1, 50 µM,24 h_ = 35, n_Mdivi-1 50 µM 24h washout after 1h_ = 27). (**c**) BN-PAGE gel shows reduced complex I activity in the gel (IGA), which is due to reduced formation of N-respirasomes (right panel)**.** IGA with separated protein from isolated mitochondria. (**d**) Complex I activity determined by NADH:DQ oxidoreduction activity after treatment with Mdivi-1 and Rotenone in isolated mitochondria of HeLa cells. (**e**) Representative docking poses with residue interactions of piericidin A and Mdivi-1 in complex I [*Mus musculus*] (6ZTQ) generated with Autodock (Hydrophobic interactions = light green, PiPi interaction = red, interaction with Tyrosine 108 = dark green, interaction with Histidine 59 = violet). (**f**) Number of interactions in best docking poses of Autodock experiment identical to the piericidin control in the inhibitor-bound cryo-EM structure. (**g**) Efficiency and binding energy of all dockings with different ligands (N = 4 docking processes, n = 25 docking poses). Boxplots indicate median (line), 25th-75th percent percentile (box) and minimum and maximum values (whiskers). Statistics: one-way ANOVA. ***p ≤ 0.001; **p ≤ 0.01; **p ≤ 0.05.

We asked whether reduced supercomplex levels in short-term treated Mdivi-1 cells was linked to inhibition of complex I by Mdivi-1^34^. To determine the effect of Mdivi-1 on the activity of complex I, respiratory activities were determined as oxygen consumption rates in the presence of substrates and inhibitors. Therefore, cells were permeabilized with digitonin and specific OXPHOS substrates and inhibitors were added. Cells were treated with Mdivi-1 (and DMSO only as control) 24 h prior to the OCR measurement (Fig. 4a). Acute Mdivi-1 treatment resulted in a significant decrease in CI + CIII/CIV electron transport activity in HeLa cells (Fig. 4b) and neurons (Supplementary Fig. S3). Treatment with 50 µM Mdivi-1 for 1 h and following washing out resulted in a significant increase of CI + CIII/CIV respiration compared to the treatment with Mdivi-1 persistent in the medium, indicating a regeneration of the CI respiration and thus reversibility of Mdivi-1 effect. The CII-dependent respiration (CII + CIII/CIV activity) was not significantly altered upon Mdivi-1 treatment compared to control HeLa cells but CIV activity was lower in Mdivi-1 treated cells. We propose, that the reduced supercomplex formation (Fig. 2) is linked to the decreased CIV activity, as supercomplex assembly enhances electron transfer efficiency due to a decreased diffusion distance of cytochrome *c*^38^. Next, a BN-PAGE with proteins from isolated mitochondria of Mdivi-1 treated HeLa cells was used for an in-gel activity (IGA) assay. Acutely Mdivi-1 treated HeLa cells displayed a lower intensity of violet bands in the gel indicating reduced CI activity (Fig. 4c) related to reduced N-respirasome formation as the quantification shows.

To test whether Mdivi-1 is a direct inhibitor of CI, mitochondria of HeLa wildtype cells were isolated. The enzyme activity of total CI was determined by time-dependent NADH oxidation with decyl-ubiquinone (DQ) as electron acceptor (Fig. 4d). Antimycin A was used to block further electron transfer to Complex III. NADH:DQ activity was measured in mitochondria of HeLa (Fig. 4d) and neuronal progenitor cells (NPC) (Fig. 4e). Mdivi-1 concentrations of at least 50 µM showed a significant decrease in CI activity indicating that Mdivi-1 is a direct inhibitor of CI. The NADH:DQ activity in the presence of 1 µM rotenone and 50 µM Mdivi-1 showed a similar inhibitory effect. To determine whether Mdivi-1 interacts in the Q-cavity or with the peripheral arm of complex I, the NADH oxidation activity of the enzyme activity was determined using ferricyanide as an electron acceptor. The NADH:FeCy oxidation of NPC mitochondria was not significantly altered by addition of 50 µM Mdivi-1 to the activity buffer (Fig. 4f). This suggests that Mdivi-1 blocks the quinone binding of CI and not the NADH oxidation site.

To test this, an *in-silico* approach was used to calculate binding energies of Mdivi-1, ubiquinone and known inhibitors of complex I. The cryo-EM structure of piercidin-binding complex I from *Mus musculus* (6ZTQ) was used as a docking platform. First, the molecular structure of the inhibitor piercidin A was manually removed from the overall structure. Different molecular docking algorithms were used for a global docking with the ligands piericidin A, Mdivi-1, rotenone, ubiquinone (Q1) and ubiquinone-10 (Q10). The best docking poses of the re-introduced piericidin A (CTRL) and Mdivi-1 in the cropped version of 6ZTQ generated by Autodock are shown in Fig. 4g. The demonstrated interactions with residues in the Q-cavity were analyzed by the number of identical interactions of piericidin A found in the original cryo-EM structure of CI co-crystallized with piericidin A. The original piericidin A contained 21 interactions, while the docking pose generated by redocking piericidin A (CTRL) contained 11 interactions. The docking of all ligands, including Q10 but not Q1, involved two essential interactions^39,40^ that are critical for binding to this CI cavity (Fig. 4h). The total number of identical interactions was higher for the ligands Mdivi-1, rotenone and Q10 compared to piericidin A, which is not a primary inhibitor of CI. In an analogous docking experiment using the CI cryo-EM structure of *Bos taurus* (5LDW), first a structural alignment was conducted to determine the homology between the two structures based on their shapes and three-dimensional conformations. Structure alignment of the full CI structures of 6ZTQ and 5LDW showed a root-mean-square deviation (RMSD) of atomic positions of 4.32 Å (Supplementary Fig. S5), which is low for a large protein complex like complex I. Docking of inhibitors to complex I from *Bos taurus* (5LDW) showed fewer interactions and lower binding energies for each ligand. The overall efficiencies and binding energies of docking calculations using Autodock algorithms and all structures of each host organism were pooled for final comparison. The mean efficiency for all docking poses determined in YASARA was significantly increased for the ligands Mdivi-1 and rotenone compared to piericidin A control (Fig. 4i) whereby Mdivi-1 showed higher binding efficiency than rotenone. On the other hand, Mdivi-1 poses showed the same binding energy as rotenone (Fig. 4i). Together with the in vitro results, these experiments indicate that Mdivi-1 acts as a local inhibitor in the Q cavity of complex I, similar to rotenone.

### Long-term treatment with Mdivi-1 significantly reduces synaptic activity in neurons

**Figure 5:**
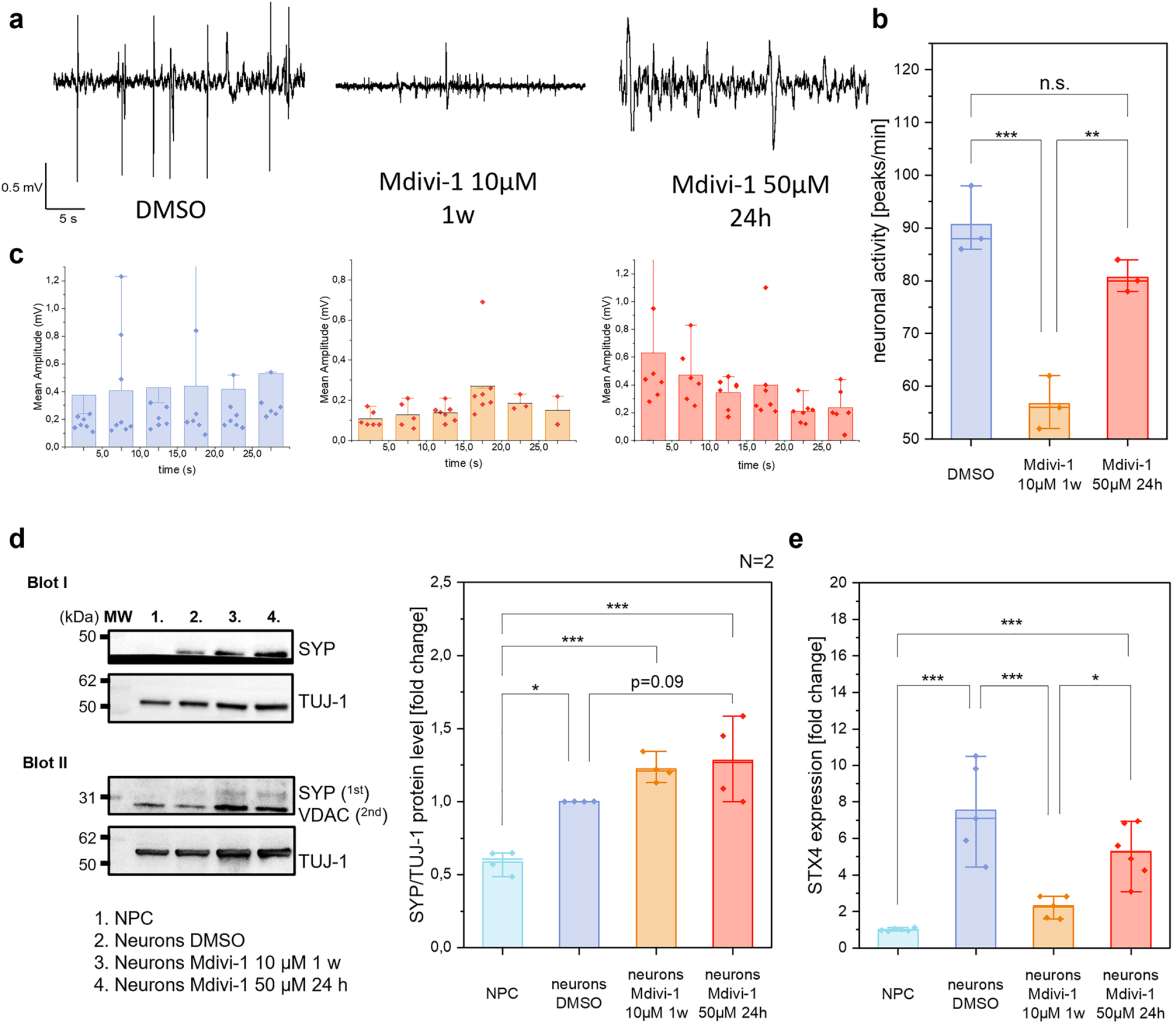
Mdivi-1 reduces neuronal function. (**a**) Exemplary MEA traces after stimulation with glutamate/glycine (100 µM each). (**b**,**c**) Peak number and amplitude in control and Mdivi-1 treated neurons (N = 2 differentiations, n = 9, ANOVA). (**d**) Quantification of synaptophysin (SYP) protein levels in control and Mdivi-1 treated NPC-derived neurons (N = 2, n = 4, ANOVA). (**e**) Gene expression of syntaxin 4 is decreased due to Mdivi-1 treatment (N = 2, n = 6, ANOVA). Boxplots indicate median (line), 25th–75th percent percentile (box) and minimum and maximum values (whiskers). Statistics: one-way ANOVA correction. ***p ≤ 0.001; **p ≤ 0.01; **p ≤ 0.05.

To determine the significance of Mdivi-1 inhibition of CI for neuronal activity, we conducted electrophysiological measurements of stimulated neurons via Microelectrode Arrays (MEA). Because of the mainly glutamatergic neuronal cell culture, pharmacological stimulation was performed with glutamate/glycine (each 100 µM) (Fig. 5a). The number of peaks per minute was significantly decreased by 38% in long-term Mdivi-1 treated neurons compared to control neurons (Fig. 5b). Acute Mdivi-1 treatment of neurons led to a non-significant decrease by 11%. Figure 5c shows the mean peak amplitude in time intervals of 5 s. Neurons with long-term Mdivi-1 treatment had a reduced mean amplitude of 0.16 mV (± 0.05 SD) compared to the DMSO mean amplitude of 0.43 mV (± 0.05 SD). Under both conditions, the amplitude is the same, while acute treatment with Mdivi-1 reduces the electrical activity of the neurons, as shown by the reduced amplitude (Fig. 5c). To test, whether vesicle fusion was involved in the deterioration of electric activity, we determined two marker proteins of the pre-synapse: synaptophysin (SYP) and syntaxin 4 (STX4). SYP is also known as the major synaptic vesicle protein p38. STX4 is part of the SNARE complex, which induces the fusion of synaptic vesicles with presynaptic terminals. Immunoblotting of SYP revealed a significant increase of the protein in differentiated neurons compared to non-differentiated NPC. Cells with acute Mdivi-1 treatment showed a tendency towards an elevated SYP level compared to DMSO treated neurons (Fig. 5d), indicating that Mdivi-1 did not affect the protein levels of SYP. However, the expression of STX4 was significantly reduced in long-term Mdivi-1 treated neurons (Fig. 5e). This could cause an impaired fusion of synaptic vesicles with the membrane after stimuli. Since vesicle fusion is Ca^2+^-dependent, we qualitatively monitored Ca^2+^-dynamics in NPC-derived neurons stained with the calcium-indicator dye Fura-2. The pharmacological activation clearly led to a reaction in the form of calcium transients, which are typical of neuronal activity (Supplementary Fig. S6). Calcium traces showed no significant difference of basal activity due to Mdivi-1 treatment, but the stimulated cells exhibited significantly less calcium uptake in neurons that were treated with 10 µM Mdivi-1 for one week during the differentiation process.

### Mdivi-1 alters the cellular and mitochondrial calcium homeostasis

**Figure 6:**
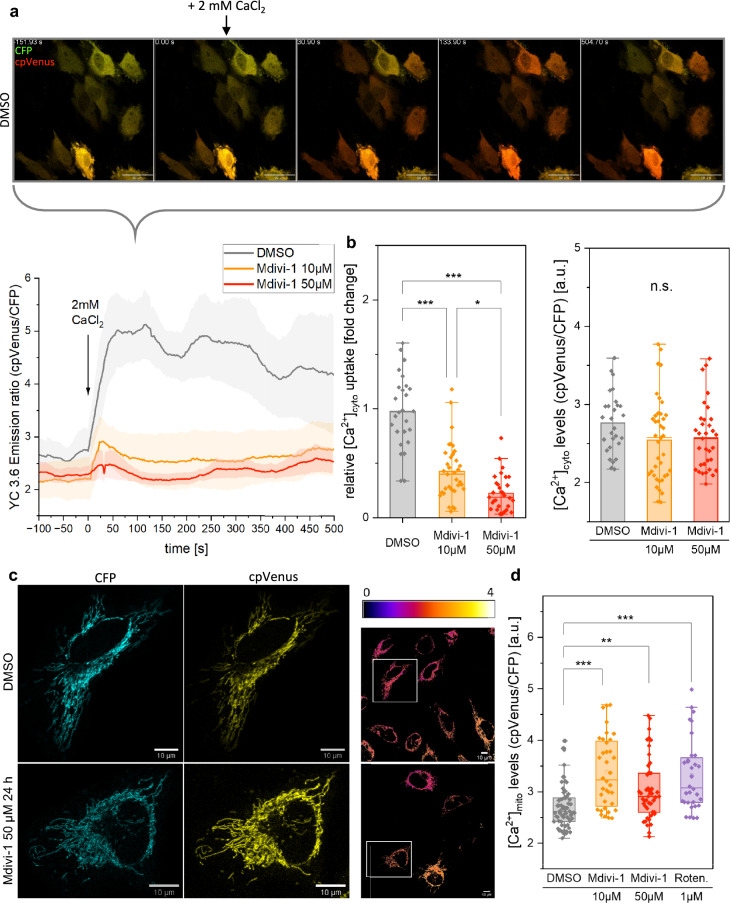
Mdivi-1 alters cellular and mitochondrial calcium homeostasis. (**a**) Representative time series of a [Ca^2+^]_cyto_ uptake experiment monitored via the YC 3.6 biosensor in transiently transfected HeLa (false color: green = CFP, red = cpVenus) and ratio-metric quantification of [Ca^2+^]_cyto_ uptake experiment with one biological replicate (scale bar: 50 µm). (**b**) Basal cytosolic calcium uptake is impaired in Mdivi-1-treated HeLa cells (N = 4, n_DMSO_ = 26, n_Mdivi-1,10 µM, 24 h_ = 35, n_Mdivi-1, 50 µM,24 h_ = 31, KW). (**c**) Exemplary images of mt4D3-cpV transfected HeLa cells (false color: cyan = CFP, yellow = cpVenus; ratio-metric image with fire LUT; scale bar: 10 µm). (**d**) Mdivi-1 treatment leads to elevated mitochondrial calcium levels (N = 3, n_DMSO_ = 67, n_Mdivi-1,10 µM, 24 h_ = 36, n_Mdivi-1, 50 µM,24 h_ = 47, n_Rotenone_ = 32, KW). Boxplots indicate median (line), 25th-75th percent percentile (box) and minimum and maximum values (whiskers). Statistics: one-way ANOVA. ***p ≤ 0.001; **p ≤ 0.01; **p ≤ 0.05.

To investigate Mdivi-1 effects on Ca^2+^ homeostasis, fluorescent FRET-based biosensors of the Chameleon family were used. The tests were exemplary done in HeLa cells. The basal cytosolic calcium level was not altered in Mdivi-1 treated cells compared to DMSO control. Cytosolic calcium uptake was studied after stimulation with extracellular calcium chloride (2 mM) (Fig. 6a). To study the Mdivi-1 effect, HeLa cells were treated with 10 µM and 50 µM Mdivi-1 for 24 h. The mean cytosolic calcium uptake was reduced by 55% in cells pretreated with 10 µM Mdivi-1 and by 75% in cells pretreated with 50 µM Mdivi-1 (Fig. 6b). To determine effects of Mdivi-1 on the mitochondrial calcium levels, cells were transfected with a calcium sensor fused to a targeting sequence for the mitochondrial matrix (Fig. 6c). The mitochondrial calcium level of Mdivi-1- and rotenone-treated HeLa cells were significantly elevated (Fig. 6d) indicating disturbed Ca^2+^-buffering by mitochondria.

### Mitochondrial morpho-dynamics is affected by Mdivi-1 treatment

**Figure 7:**
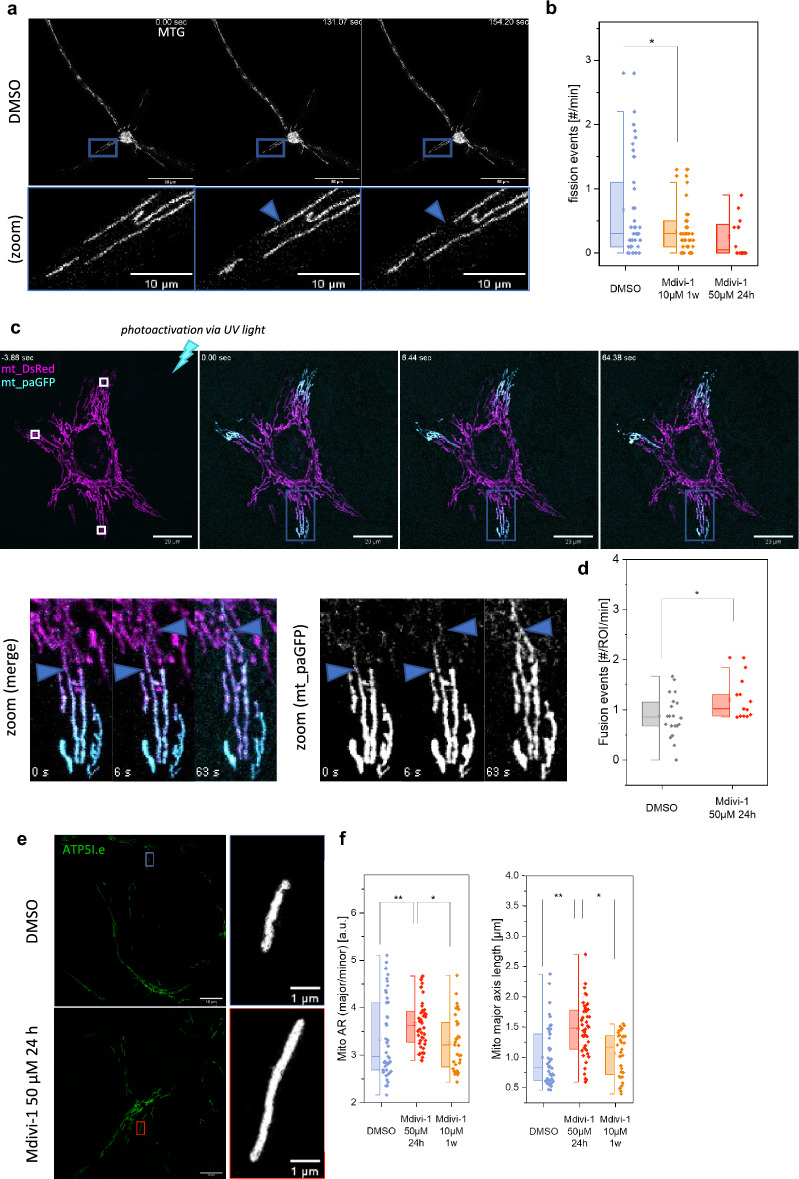
Mdivi-1 effects on mitochondrial morpho-dynamics. (**a**) Exemplary image series of NPC-derived neurons stained with MTG (scale bar: 50 µm), lower panel shows zoomed regions of interest with blue arrows indicating two fission events (scale bar: 10 µm). (**b**) Quantification of fission events in Mdivi-1 treated neurons (N = 2, n_DMSO_ = 39, n_Mdivi-1 10 µM 1w_ = 39, n_Mdivi-1 50 µM 24 h_ = 12). (**c**) Time course showing mitochondrial dynamics in HeLa cells after photoactivation of mt-paGFP, mitochondria were co-transfected with mt-dsRed and mt-paGFP (scale bar: 20 µm) as described in (Molina and Shirihai 2009). (**d**) Number of fusion events in similar sized regions of interest. (**e**,**f**) Mitochondrial morphology analysis in neurons. (**e**) Structured illumination microscopy of immuno-stained mitochondria. (**f**) Quantification of the aspect ratio (AR), length of mitochondria and mitochondrial perimeter. Boxplots indicate median (line), 25th–75th percent percentile (box) and minimum and maximum values (whiskers). Statistics: Kruskal–Wallis-ANOVA. ***p ≤ 0.001; **p ≤ 0.01; **p ≤ 0.05. Scale bars: 10 µm (A), 20 µm (**c**) and 1 µm (**e**).

Finally, we asked whether Mdivi-1 treatment would affect mitochondrial morphology and dynamics as reported in previous work^28^. Neuronal mitochondria were stained with MitoTracker™Green (MTG) and imaged via structured illumination microscopy, SIM (Fig. 7a). Neurons treated with Mdivi-1 for one week displayed decreased fission events (*p* ≤ 0.05). Treatment with higher concentration of Mdivi-1 but for shorter time induced no difference in fission rates (Fig. 7b). To determine fusion, we used mitochondria-targeted photo-switchable GFP in combination with MitoTracker™Deep-red staining (Fig. 7c). paGFP was activated with UV-light in several regions of interest. The spreading of the mitochondrial green fluorescence allowed for the quantification of fusion events. We found a slight increase in fusion rates after 1 day treatment (*p* ≤ 0.05) (Fig. 7d). To determine morphological parameters of mitochondria in neurons, we immune-stained mitochondria with an antibody against ATP synthase subunit ATP5Ie and imaged the mitochondria by structured illumination microscopy (Fig. 7e). We found an increased aspect ratio (length/width) of individual mitochondria, the mitochondrial axis was longer and the perimeter increased in Mdivi-1 treated cells (Fig. 7f).

## Discussion

The Mitochondrial Division Inhibitor-1 Mdivi-1 has been discussed as a potential therapeutic treatment for neurodegenerative diseases^41^. However, the mechanism of action is still not fully understood and recent data questioned its effect on Drp1-mediated organelle fission and suggested an inhibitory effect on bioenergetics, in particular complex I activity^34,35,42^. We here showed in detail that Mdivi-1 is an inhibitor of respiratory complex I similar as rotenone. Mdivi-1 treatment resulted in reduced CI activity and oxygen consumption rates. Inhibition of complex I by Mdivi-1 affects respiratory complex supercomplex formation and decreased mitochondrial ATP levels, resulting in a reduced mitochondrial function. Together with an altered calcium dynamics finally resulted in impaired neuronal activity.

As a mechanism of action, we showed that Mdivi-1 specifically inhibits the ubiquinone reduction in respiratory CI (I_Q_-site), while NADH:FeCy oxidation was not affected. NADH:DQ oxidations rates were reduced in Mdivi-1-treated mitochondria. The Q-cavity is a target of a variety of structurally different CI inhibitors, in particular of rotenoids (e.g. rotenone)^43^. The potency of rotenoids to bind to the I_Q_-side is due to a specific spatial organization of hydrogen-bond acceptable methoxy oxygens that allow the tight fitting into the binding site and provide a bending axis between thermodynamically stable phenol rings. The structure of Mdivi-1, chemically 3-(2,4-dchloro-5-methoxyphenyl)-2-sulfanyl-4(3H)-quinazolinone, contains three aromatic rings which are also not in one plane. The bond between the quinazoline moiety and the phenyl can be twisted, a prerequisite for stable positioning of Mdivi-1 in the I_Q_-side of Complex I (Fig. 4g). The oxygen atom of the hydroxyl group and the chloride ion of the first aromatic ring in Mdivi-1 provide similar binding possibilities as the two methoxyl groups on the A-ring of rotenone. The computational interaction analysis revealed additional hydrogen bonds Met69 (NDUFS7), Met70 (NDUFS7), Phe86 (NDUFS7) and Thr156 (NDUFS2) that are reported to further stabilize the inhibitors in the binding site^39^.

Since 50 µM Mdivi-1 had comparable effects to 1 µM rotenone, one of the strongest inhibitors of the I_Q_-binding site (Fig. 4h), a higher *K*_I_ of Mdivi-1 can be assumed. Mdivi-1 exclusively inhibited CI, since CII-dependent respiration was not altered. As a further effect, ROS levels increased. These effects are similar as it was earlier observed for rotenone^44^. Increase of ROS upon inhibition of CI is typical for A-class type inhibitors of the I_Q_-site of CI^45,46^. It was suggested that binding of Mdivi-1 to CI is reversible^34^, but we found only partial regeneration of CI-dependent respiration after removal of Mdivi-1. We assign the incomplete regeneration to the observed CI degradation and supercomplex (SC) disassembly, respectively, as discussed in the following paragraph.

### Potential mechanisms for the discovered alterations in SC formation

Acute Mdivi-1 treatment decreased the formation of respiratory SC containing CI, the N-respirasomes, but also SC without CI, the Q-respirasomes. The relative expression of SC assembly factor HIGD2A was significantly decreased by Mdivi-1 treatment. HIGD2A promotes the assembly of CIV by adding the Cox3 module but also associates with I + III_2_ supercomplexes and adds complex IV, leading to the formation of a respirasome^14^. Reduced interaction between SC I + III_2_ and IV and VI_2_, respectively, can explain the increase of I + III_2_ compared to respirasomes as shown in the SC line plot of Fig. 2a. Transcriptional regulation of HIGD2A function is a regulator of respiratory supercomplexes assembly in response to hypoxia, cellular metabolism and cell cycle: knock out of HIGD2A in the murine skeleton muscle C2C12 cell line resulted in less SC, higher OPA1 levels, increased [ROS]_mito_ and increased ΔΨ_m_^47^. These effects match our findings. It is suggested, that SCAFI stabilizes SC without CI ^48^. The observed protein level of the assembly factor SCAFI was not significantly decreased, however the standard deviation in between replicates of the immunoblotting of SCAFI was very high and the total protein level does not show how much SCAFI protein is bound in the assembled supercomplexes. In contrast to the decreased SC without CI, the mRNA expression of SCAFI was increased in HeLa cells, which might be a compensation due to the decrease of all supercomplexes. A SC form denoted with [I + III_2_]_2_ of higher molecular weight was observed at ≈ 1300 kDa showing either a respirasome with a trimer of CIV (I + III_2_ + IV_3_), or I_2_ + III_2_^49^ or the human mitochondrial megacomplexes^37^. To further dissect the exact composition of complexes in the quantified band, quantitative mass spec analysis would be required.

The separation of mitochondrial complexes by BN-PAGE and subsequent immunoblotting of complex I subunits NDUFS3 and NDUFB10 revealed an increase of an intermediate form with a molecular weight of approximately 850 kDa (Figs. 2, 3). Monomeric complex I is hardly found in human mitochondria. Therefore, the intermediate form can be either the smallest form of an SC that lacks the last assembly factors (pre-I + III_2_)^50^, an incompletely assembled CI with a molecular weight of ≈ 830 kDa^51^, or a respirasome subcomplex that contains incompletely assembled CI. The following sections will discuss potential reasons for the increase of this intermediate form and will also discuss the different pathways for CI assembly.

### CI perturbation reduces the level of N-respirasomes

Recent studies have shown that knockout of NDUFB10, which is an accessory subunit of CI stabilizing the P-arm of the complex, results in incomplete assembly of CI^52,53^. The loss of subunits of the N- and P-module ultimately led to the loss of CI and respiratory supercomplexes. Assembly analysis of the CI-containing supercomplexes in a study with different knockout cell lines of CI accessory subunits (e.g. NDUFA8-KO, NDUFS5-KO, NDUFC1-KO) revealed the loss of supercomplexes by BN-PAGE^53^. Those studies provide evidence that fully assembled CI is required for SC formation, as previously described^50^. Furthermore, it is suggested that CI assembles in the absence of CIII but is unstable, and inhibition of CIII activity does not affect CI assembly^18^. Since an intermediate form of CI (similar to pre-CI proposed in^51^) was found in Mdivi-1 treated cells, this led us conclude that impairment in CI assembly results in a decrease of supercomplexes.

### The decrease of supercomplexes (with and without CI) leads to CI destabilization

The NDUFB10-KO as well as a ND6-KO cell line of CI completely prevented the formation of N-respirasomes, but still allowed the formation of the supercomplex III + IV^12^. Here, we found also a decrease of supercomplexes without CI (Q-respirasome). The cooperative model suggests that CI builds up as a pre-CI of ≈ 830 kDa, with the binding site of the N-module subunit being occupied by NDUFA12 to stabilize the pre-CI. In addition, CIV associates with CIII, which in turn binds to the pre-CI^54^. Finally, NDUFA12 is exchanged to assemble the N-module to provide a functional respirasome. According to this model, the impaired connection of CIII with CIV can hinder the biogenesis of CI. This argumentation is also relevant for another proposed assembly pathway, which describes a similar cooperative model, but an earlier association of CIII with a membrane arm of CI^17^. A decrease of N-respirasomes would decrease the integrity and stability of CI. The N-module is assembled as the last step in these two models, but the observed intermediate form shows slight NADH-oxidation by CI in-gel activity (Fig. 4c), which suggests that the destabilized CI form still contains the N-module. A decrease of all SC forms increased superoxide production.

CI inhibition leads to a conformational change in the protein, altering the closed state and the angle of the membrane arm to the peripheral arm, and additionally preventing its function. Persistent binding of Mdivi-1 to CI could therefore alter the interaction with CIII and CIV and thus contribute to N-respirasome disassembly and CI instability. Whether CI inhibition directly affects CI assembly or SC formation has not been reported in the literature and was not further investigated in this study.

### Inhibition alters morpho-dynamics of mitochondria towards an elongated shape

Since Mdivi-1 was introduced as the mitochondrial division inhibitor-1, we examined mitochondrial dynamics and morphology in neurons and non-polar cells. Long-term treatment induced mitochondrial elongation due to an imbalance in fusion and fission towards more fusion. Mitochondrial elongation is as stress response that can be neuroprotective^55–57^, but we still found impairment of neuronal activity.

### Long-term Mdivi-1 inhibition of CI and disturbance of ETC function impairs neuronal activity

Long-term Mdivi-1 treatment during differentiation resulted in reduced electrical activity of neurons, as exhibited in fewer electric spikes per minute using a MEA assay (Fig. 5a). Mitochondria control neuronal activity mainly by providing ATP and mediating calcium signaling required for vesicular exocytosis, endocytosis and vesicle recycling, as well as for powering synaptic transmission. The impaired ETC activity due to decreased CI activity and reduced N- and Q-respirasome levels resulted in decreased ATP production. The reduced efficiency of ATP production eventually led to energy starvation of the neurons. We further found that long-term treatment with Mdivi-1 (10 µM, 1 week) resulted in an attenuated level of cytosolic calcium in HeLa cells and neurons that were stimulated with glycine/glutamate (Fig. 6B and Supplementary Fig. S6). This can also be related to decreased ATP, be due to an impaired function (or decreased protein level) of neuronal Voltage-gated calcium channels (VGCCs) or be due to an altered intra-cellular calcium buffering. Indeed, we found that [Ca^2+^]_mito_ levels were increased in HeLa cells. We assume that mitochondrial calcium buffering is also increased in neurons and thus the calcium homeostasis and dynamics of neurons is altered due Mdivi-1 treatment, which is in line with a previous report^35^. This study showed that short Mdivi-1 treatment (50 µM, 1h) induced a reduction of cellular and mitochondrial Ca^2+^ uptake, when cells were exposed to NMDA or AMPA/CTZ.

Another reason for the reduced activity of long-term Mdivi-1-treated neurons may be the impairment of synapse formation at a later stage of differentiation. We found no change of the total presynaptic vesicle-related SYP protein level after short- or long-term Mdivi-1 treatment, though (Fig. 5d). However, we found a reduced expression of syntaxin 4 (Fig. 5e). Syntaxins bind synaptotagmin in a calcium-dependent fashion and interact with voltage dependent calcium and potassium channels. Direct syntaxin-channel interaction links the vesicle fusion machinery and the gates of [Ca^2+^]_cyto_ entry during depolarization of the presynaptic axonal boutons^58^. Our current hypothesis is that long-term Mdivi-1 treatment leads to reduced neuronal function due to impaired calcium-dependent vesicle fusion, which reduces exocytosis.

## Conclusion

Our data show that inhibition of mitochondrial complex I by Mdivi-1 destabilizes not only the complex but also entire respirasomes. This results in decreased ATP production, disturbed Ca^2+^ homeostasis and eventually neuronal dysfunction. Mitochondrial elongation as a stress response could not counteract these impairments. In view of the results presented here, a possible therapeutic application of Mdivi-1 must take into account these dose- and time-dependent effects on mitochondrial energy and calcium metabolism.

## Material and methods

### Cell lines

Hela cells were purchased from the Leibniz Institute DSMZ-German Collection of Microorganisms and Cell Cultures (#AC 57) and maintained in supplemented MEM-medium (Minimum essential medium Eagle, Sigma-Aldrich, M2279; 10% FBS supreme, PAN BioTech P30-3031; 1% HEPES, Sigma-Aldrich H0887-100ML, Ala-Gln, Sigma-Aldrich G8541-100ML, 1%; MEM non-essential amino acid solution, Sigma-Aldrich, M7145-100ML, 1%) following usual protocols.

Neuronal progenitor cells (NPC) were a kind gift of Prof. Thomas Gasser, Neurologische Universitätsklinik Tübingen, Germany. NPC were cultured on 1% [(v/v) in KO-DMEM/F-12, Thermo Fisher] matrigel (BD Biosciences)-coated 6-well plates (Sarstedt) in Neuronal Keeping Medium (NKM), composed of N2B27 medium with addition of the small molecules smoothened agonist (SAG, 0.5 µM, Cayman Chemical) and CHIR 99021 (3 µM, Axon MedChem) and 150 µM ascorbic acid (Sigma-Aldrich).

The differentiation protocol is depicted in supplementary Fig. S1. It generates midbrain NPC-derived neurons.

### Cell transfection

Hela cells were transfected with the reagent Polyethylenimine (PEI, Polysciences Inc.) and NPCs were transfected with TurboFectin 8.0 from OriGene. The best transfection efficiency (≈ 0.001%) of NPC in a 12-well plate was found using 0.8 µg of total plasmid DNA.

### Mitochondrial mass and morphology

MitoTracker™Green FM (MTG) is a membrane potential-independent mitochondrial tracker with excitation/emission maxima ∼490/516 nm, which accumulates in mitochondria and binds covalently to mitochondrial proteins by reacting with free thiol groups of cysteine residues. However, a minimum amount of membrane potential is needed to allow the incorporation of the dye. MTG (Invitrogen, #M7514) was used to stain mitochondrial mass for Mitochondrial Network analysis and normalization of dyes monitoring specific bioenergetic parameters. Cells were stained at a final concentration of 100 nM for 30 min at 37 °C and 5% CO_2_. Next, one washing step with PBS and two washing steps with culture medium were performed on HeLa cells to remove cytosolic background signal. For neurons, staining medium was aspirated carefully and three washing steps with NDM were performed prior to imaging with the cLSM.

### Mitochondrial ATP levels

ATP-Red Live cell dye (Biotracker) reports mitochondrial ATP levels, when a negatively charged ATP breaks the covalent bonds between boron and ribose, causing ring-opening and fluorescence. The red fluorescent dye has excitation/emission maxima of ∼ 510/570 nm. Cells were incubated with 5 µM ATP-Red for 15 min at 37 °C. Cells were simultaneously stained with MTG for normalization on the mitochondrial mass. Next, washing steps were performed as previously described for MTG staining and fresh medium was added prior to imaging.

### Mitochondrial superoxide levels

MitoSox (Thermo Scientific) detects superoxide localized in the mitochondria. Cells were stained with 2.5 µM MitoSox for 30 min at 37 °C and then washed three times with medium before imaging. As a control for lipid peroxidation, a sample was treated with 300 µM Tert-Butyl Hydroperoxide (TBH70X, Luperox) for 30 min.

### Immunostaining

Fixation, permeabilization and immune-staining was performed according to established protocols. Antibodies are listed in Supplementary Tables 2 and 3.

### Mdivi-1 inhibition assays

Mdivi-1 [Sigma-Aldrich M0133] was dissolved in dimethyl sulfoxide (DMSO). Acute cell treatment was performed by incubating cells for 24 h in media containing 50 µM Mdivi-1, or long-term cell treatment for 1 week in 10 µM Mdivi-1. Control experiments were performed with the same amount of DMSO.

### SDS-PAGE and blotting

Protein separation in SDS-PAGE was performed according to established protocols. Before loading, cell lysate was boiled at 95 °C for 5 min. For SCAFI immune-blotting the sample had to be shaken at 40 °C for 10 min after addition of 4 × SDS sample buffer. Equal protein quantities (HeLa 50 µg, NPC 50 µg, neurons 40 µg) were loaded on a 4%/12% gel. The separated proteins were transferred onto polyvinylidene difluoride (PVDF) membranes by wet blotting at 25 V overnight.

### Immunoblotting and membrane imaging

Immunodetection of proteins was performed according to established protocols. For detection, an enhanced chemiluminescent substrate (ECL) consisting of a 1:1 mix of Luminol/Enhancer & Peroxide solution was added to the membrane and incubated for 2–5 min. Finally, band signal detection was conducted with the ChemiDOC Infrared Imaging System (BioRad).

### Isolation of mitochondria

For isolation of mitochondria from cultured mammalian cells, ~ 23 × 10^6^ cells were harvested and cell pellets were frozen at − 80 °C. All following procedures were performed on ice. The cell pellet was briefly thawed and recentrifuged at 300 × *g* for 5 min at 4 °C. The supernatant was discarded and the pellet was resuspended in mitochondria isolation buffer (MIB) (0.32 M sucrose, 1 mM EDTA, and 10 mM Tris–HCl, pH 7.4). For mechanical destruction, the cell suspension was transferred into a Dounce homogenizer (B.Braun). The cell lysates were centrifuged at 800 × *g* for 5 min at 4 °C. This step was repeated until no cell pellet was visible any more. Finally, the supernatant was centrifuged at 10,000 × *g* for 10 min at 4 °C followed by resuspension of the pellet in mitochondrial isolation buffer and concentration quantification.

### BN-PAGE and blotting

Mitochondrial membrane protein complexes were separated using native gel PAGE as described earlier^59^. All steps were performed on ice. Mitochondrial pellets were resuspended with 6 g Digitonin/g protein. The suspension was centrifuged for 20 min at 20,000 × *g* and the supernatant with the solubilized mitochondrial membrane proteins was supplemented with 20% Glycerol and 25% detergent with Coomassie. 50 µg protein was loaded per lane and separated on a 3–13% acrylamide gradient gel. The separated protein complexes were electro-blotted onto Hybond-P-polyvinylidene fluoride (PVDF) membranes (GE Healthcare) using a wet blotting system at 25 V overnight.

### Complex I In-Gel Activity Assay (CI-IGA)

To analyze complex I activity, the blue native gel was incubated for 24 h in 20 ml complex I substrate solution until violets bands were clearly visible indicating active complex I. The reaction was stopped by denaturing the native complexes with 10% acetic acid solution. Finally, the gel was washed with water and a picture was taken.

### Enzyme activity of isolated OXPHOS complexes by spectrophotometry

Isolated mitochondria were frozen at − 80 °C, thawed on ice, resuspended briefly and refrozen to disrupt the mitochondrial membrane. Assays were performed in 96 well plates in base buffer BICA (250 mM Sucrose, 10 mM Tris/MOPS, 1 mM EGTA) with 20 µg of isolated mitochondria per well. Finally, the optical density (OD) was measured in 12 s intervals using a Cytation 5 (Agilent) in the kinetic readout mode. For the NADH:DQ activity assay, the 6-decyl derivative of ubiquinone (DQ) was added as electron acceptor, and the electron transfer from CI to CIII was blocked by inhibition of with Antimycin A (4 µM). The spectrophotometric measurement of NADH:DQ oxidation at OD 340 was performed in NADH:DQ activity buffer (130 µM NADH, 130 µM DQ, 4 µM Antimycin A in BICA) at 35 °C for 5 min. The spectrophotometric measurement of NADH:FeCy oxidation at OD 340 was done in NADH:FeCy activity buffer (130 µM NADH, 1 mM FeCy, 4 µM Antimycin A in BICA) at 32 °C for 8 min ^60^.

### Determination of gene expression via quantitative PCR (qPCR)

Total RNA of cell pellets was obtained with the Monarch Total RNA Miniprep Kit (NEB #T2010S), and cDNA was transcribed with the RevertAid First Strand cDNA Synthesis Kit (Thermo Scientific #K1621). Quantitative PCR (qPCR) was performed on the StepOnePlus Real-Time PCR System (Applied Biosystems). The PCR reaction was prepared from PowerUP SYBR Green Master Mix (Applied Biosystems # A25741), 50 ng cDNA, and 0.1 nM of each forward and reverse primer (purchased from Eurofins Genomics) per sample (Supplementary Table 1). *β*-V Tubulin was used as a housekeeping gene and ∆*C*_*T*_ was normalized to a DMSO control of the according cell type. Normalization of ∆*C*_*T*_ on untreated NPC was performed when the relative gene expression of multiple samples was investigated to observe changes throughout the differentiation process.

### Oxygen consumption measurements

All measurements of oxygen consumption rates (OCR) and extracellular acidification rates (ECAR) were performed with an extracellular flux analyzer (Seahorse XF96, Agilent) according to standard protocols.

To determine basal, ATP-synthase dependent, maximal and non-respiratory oxygen consumption, oligomycin (1 µM), Carbonylcyanid-p-trifluoromethoxyphenylhydrazon (FCCP, 0.5 µM for HeLa and 0.75 µM for neurons), rotenone (1 µM), and Antimycin A (AA, 1 µM) were added subsequently. With the final injection, the cells were stained with Hoechst 33,342 (2 µg/ml) to determine the cell number after finishing the assay (Cytation 5, Agilent).

Activities of individual respiratory chain complexes were determined after permeabilization of membranes with digitonin and application of specific OXPHOS substrates in MAS buffer (220 mM mannitol, 70 mM sucrose, 10 mM KH_2_PO_4_, 2 mM HEPES, 1 mM, EGTA, 5 mM MgCl_2_, 0.5 µM FCCP and 0.0006% digitonin, pH 7.4). The digitonin concentration was chosen at the lower end of the recommended digitonin range for permeabilization of HeLa cells or neurons, which is 0.00025–0.0025%, to protect mitochondrial membranes from cytochrome c release^61^.

To measure CI/CIII/CIV- or CII/CIII/CIV-driven oxygen consumption, either glutamate (10 mM) and malate (10 mM) as CI substrates or succinate (10 mM) and glycerol-3-phosphate (G-3-P, 5 mM) as CII substrates were added. Next, rotenone (0.5 µM) and AA (0.5 µM) were added to block CI and CIII. Finally, tetramethyl-p-phenylenediamine (TMPD, 100 µM) and ascorbate (10 mM) were injected for determination of CIV-dependent oxygen consumption alone. Measurements were done at 37 °C.

### Mitochondrial fusion analysis

Mitochondrial fusion assays were carried out as previously described^62^. HeLa cells were transfected with a paGFP fused to a mitochondrial matrix targeting sequence and mt DsRed. Live cell imaging of HeLa cells was performed at a Leica cLSM (SP8) at 37 °C with 5% CO_2_. Photoactivation of mt paGFP was achieved by laser illumination at 405 nm in 3 × 3 µm^2^ regions of interest (ROI) of mitochondria in the periphery of transfected cells. After photoactivation, we measured the green fluorescence intensity of the activated mt paGFP in the whole cell after excitation with the 488 nm laser line over 400 s with a frame rate of 0.4 Hz via photomultiplier tube (PMT) detectors. The paGFP molecules spread towards the already connected mitochondria surrounding the ROI post photoactivation, and mt paGFP-activated mitochondria may also move or fuse with close- standing mitochondria, hence leading to a decrease in fluorescence intensity within the photoactivated ROI over time as shown in Fig. 7A. The fluorescence intensity decays in the photoactivated ROI. The fluorescence intensity (I) was normalized to the initial fluorescence intensity in the first frame after activation. Due to spreading, the intensity of mt paGFP fluorescence decreased. This is shown as the overall decay in the first 400 s, and the slope of the resulting curve in the first 50 s. Since the decay is approximately linear in this region, this was measured as the delta fluorescence intensity divided by the time (∆I/∆t) as described in^63^. Furthermore individual fusion events were determined by the spreading of mt paGFP between a donor mitochondrion, with a high green fluorescence intensity and an acceptor mitochondrion, with a lower fluorescence signal. Fusion events were exclusively counted, if the red fluorescence intensity did not change significantly to prevent errors by loss of the confocal plane. The number of fusion events is described as the per minute per number of photoactivated ROI.

### Mitochondrial dynamics analysis with MitoMeter

Analysis of mitochondrial dynamics was performed by live cell imaging of MTG stained neurons. Imaging of was performed at a Leica cLSM (SP8) at 37 °C with 5% CO_2_. An image series of 2–5 cells was acquired for 10 min in intervals of 7 s with at least 1.1 × zoom as shown in Fig. 7A.

For image series without loss of focus, cellular ROIs were set for data analysis based on single cells. The recently published plugin MitoMeter for MatLab was used for automated segmentation and tracking of mitochondria^64^. The pixel size, in microns per pixel as well as time in between frames was applied for each image series for time-based analysis with equal scaling. Manual control of randomly labeled objects was performed to prevent non-mitochondrial objects created by the automated segmentation. A track length threshold of 4 was applied and only confident tracks were taken into account. Track parameters were saved and the analysis was focused on the mitochondrial speed per mitochondrion and mitochondrial fission events per minute per cell. Fission between existing tracks and a newly created track is determined by comparisons of the area and of the extrema distances of nearby mitochondria before and after fission^64^.

### Microelectrode array (MEA) measurements

Electrophysiological characterization and neuronal activity of neurons were performed by using 9-well on microelectrode array (MEA) chips (USB-MEA256system, Multichannel Systems) as previously described^65^. Cells were grown on MEA chips for about 96 h. Electric field potentials were first recorded at 37 °C under basal conditions without any activator or inhibitor to record a reference signal. To identify neurotransmitter responsive network activity, different pharmacological agonistic and antagonistic modulators were applied to each sample chamber of a MEA chip and electric field potential was recorded. The activators glutamate/glycine (100 µM each, Sigma-Aldrich), dopamine hydrochloride (10 µM, Sigma-Aldrich) and GABA (10 µM, Sigma-Aldrich) were applied to selectively detect neurotransmitter responsive neuronal networks. After recording the activator responsive signals, selective inhibitors for the respective pathways were applied to the same well. The used inhibitors were risperidone (10 nM, Sigma-Aldrich), bicuculline (1 µM, Sigma-Aldrich) and ketamine (10 µM, Sigma-Aldrich). For recording the datasets, Cardio2D software (Multi Channel Systems MCS GmbH, Reutlingen, Germany) was used. Data was analyzed using the software Cardio2D + (Multi Channel Systems MCS GmbH, Reutlingen, Germany) and Origin v9.0 (OriginLab Corporation, Northampton, MA, USA). MEA analysis was performed on n = 3 independent replicates.

### Imaging

For imaging, a cLSM (TCS SP8 SMD) equipped with a 60 × objective (NA 1.35, N/0.17/FN26.5, Leica), two Hybrid GaSP-detectors (HyD) and a tunable white-light laser was used. Live cell imaging was performed at 37 °C and 5% CO_2._

### Calcium imaging

Cytosolic uptake was measured by ratio metric imaging of Yellow Chamelaeon YC 3.6-transfected HeLa cells via stimulation with 2 µM CaCl_2_ for 8 min after recording a baseline of 2 min. Mitochondrial Calcium ([Ca^2+^]_mito_) levels were measured with the Chameleon biosensor mt4D3cpv, which contains a targeting sequence for the mitochondrial matrix and a circular permuted Venus as an acceptor fluorophore for FRET. Cytosolic Calcium concentrations were measured by F340/380 ratio metric imaging of Fura-2 AM. Cells were stained with 5 µM Fura-2 AM and calcium transients were recorded with a Nikon Eclipse Ti–S microscope equipped with a 40X oil immersion objective with 0.5 frames per second in the MetaFluor Fluorsecence Ratio Imaging Software. Basal and stimulated neuronal activity was determined by cellular calcium uptake pre and post activation with glutamate/glycine (each 100 µM). Measurement of neuronal calcium uptake was quantified by the area under curve (AUC) starting from the baseline.

### In silico Molecular Docking experiments with complex I

All structural alignments and docking experiments were performed by using YASARA structure 2023 via implemented and adapted macros. The previously published inhibitor-bound complex I cryo-EM structure obtained from *Mus musculus* with PDB number 6ZTQ^39^ was used as a docking platform after removing the inhibitor piercidin A. Complex I from *Bos taurus* (PBD: 5LDW)^66^ and from *Homo sapiens* (PBD: 5XTD)^37^ were used for further experiments with respiratory complexes of other hosts. Prior to docking, the four ligand structures of rotenone, Mdivi-1, Q1 and Q10 were imported as SMILES strings in YASARA and energy minimized. Next, the previously cropped piercidin A was added to the ligands as a control. Global docking was performed using the previously described cryo-EM structures with a simulation box surrounding the Q-cavity of complex I as binding site and the modified YASARA macro dock run.mcr with 100 runs per ligand. Additionally, cryo-EM structures were cropped around the binding domain for docking experiments with AutoDock. For subsequent analysis of interacting residues, the docking conformation with highest binding energy was chosen for each ligand and compared to the interactions in the inhibitor-bound cryo-EM structure. N = 4 docking processes with n = 25 docking poses each were performed. According to recent publications^39,40^ and specific residues were identified as crucial for ligand interactions in the Q-cavity and therefore focused on during analysis.

### Statistical analysis

All graphs were generated with Origin v9.0 (OriginLab Corporation, Northampton, MA, USA). Pooled data of biological replicates were checked for significant outliers via Gubbs test. After removal of significant outliers, statistical analysis for normally distributed data was performed via one-way ANOVA with Tukey post-hoc tests, while not normally distributed data was analyzed via Kruskal–Wallis-ANOVA with Dunn’s post-hoc tests. Asterisks indicate level of statistical significance: *p ≤ 0.05, **p ≤ 0.01, ***p ≤ 0.001, tendency *p* ≤ 0.1 or not significant *p* > 0.05. *N* indicates the number of independent experiments performed with a different batch of cells or for differentiated neurons a new differentiation and *n* represents the number of total measurements.

### Cells

The NPC were a gift from Thomas Gasser, Universitätsklinikum Tübingen, Germany. HeLa cells were purchased from the DSMZ (Leibniz Institute, German collection of Microorganisms and Cell Cultures GmbH, #ACC57).

### Supplementary Information

Supplementary Information 1.

Supplementary Information 2.

Supplementary Information 3.

Supplementary Information 4.

Supplementary Information 5.

Supplementary Information 6.

Supplementary Information 7.

Supplementary Information 8.

Supplementary Information 9.

Supplementary Information 10.

Supplementary Information 11.

Supplementary Information 12.

Supplementary Information 13.

Supplementary Information 14.

## Data Availability

This study did not generate new unique reagents nor genetically modified organisms.

## Data availability

The datasets used and/or analysed during the current study available from the corresponding author on reasonable request.
