# Supplementary material for: Detailed analysis of Mdivi-1 effects on complex I and respiratory supercomplex assembly
Source: Sci Rep. 2024 Aug 24;14:19673. doi: 10.1038/s41598-024-69748-y (PMC11347648; doi:10.1038/s41598-024-69748-y)
Supplement: Supplementary file 1 — Supplementary Information 1. [file 41598_2024_69748_MOESM1_ESM.docx]

# Detailed analysis of Mdivi-1 effects on complex I and respiratory supercomplex assembly

## Supplementary Material

Table 1: List of used primers for quantitative PCR (qPCR).

**Gene Forward (5’** → **3’) Reverse (3’** → **5’)**

**Signaling**

PGC1alpha CCA AAG GAT GCG CTC TCG TTC A CGG TGT CTG TAG TGG CTT GAC T

TFAM TCC TCC CTC TTT CAT TCT CC AGA CTC CAA GCA CTT TAC AAC

**Supercomplex formation**

HIDG1A CAG ACA CAG GTG TTT CCC TTC C AGC CCA TAC CAA CAG TCA TTG C

HIDGD2A AAG TTC GTT CGC AAG ACC CG TCA TAG CAG TGA CAG CCA GAC C

SCAFI CACCAACTAAACTGACCTCCG GGGCACACCATCAGCTTTCT

**OXPHOS subunits**

ND1 CTA CTA CAA CCC TTC GCT GAC GGA TTG AGT AAA CGG CTA GG

ND4 CTA GGC TCA CTA AAC ATT CTA CTA GGC TCA CTA AAC ATT CTA

NDUFA9 AGC TTC ATC ATG CCC TCA TGC C TCT CGC GTC CCA TTC CAG AAA C

SDHA GCA AAA TCA TGC TGC CGT GTT C ATC CGC ACC TTG TAG TCT TCC C

UQCRC1 GAG CAC CAG CAA CTG TTA GAC C CAG TGA AGC GGC ATG GAG TAA G ATP5F1B GAA GAC AAG TTG ACC GTG TCC C TCA CGA TGA ATG CTC TTC AGC C

**Neuronal markers**

SOX-2 GCTACAGCATGATGCAGGACCA TCTGCGAGCTGGTCATGGAGTT

OCT-4 CCTGAAGCAGAAGAGGATCACC AAAGCGGCAGATGGTCGTTTGG

GAP43 GAGCAGCCAAGCTGAAGAGAAC GCCATTTCTTAGAGTTCAGGCATG

MAP-2 TGG CAT TGA CCT CCC TAA AGA G TTG CTT CCG TTG GCA TTT CG

STX4 CGG CAG ACT ATT GTC AAA CTG GG ATC TCA TCG CGC AGG TTC TGC A

Table 2: List of used primary antibodies for Immunofluorescence (IF) and Immunoblotting (IB).

| **Primary Antibody** | | **Manufacturer** | | **source** | | **Dilution (IF)** | | **Dilution (IB)** | |
| --- | --- | --- | --- | --- | --- | --- | --- | --- | --- |
| Anti-ATP5i | | abcam (ab12241) | | rb | | 1:200 | | - | |
| Anti-CHAT | | Proteintech (20747-1-AP) | | rb | | 1:200 | | - | |
| Anti-MAP2 | | Invitrogen (PA5-17646) | | rb | | 1:400 | | - | |
| Anti-MTCO1 | | Invitrogen (459600) | | ms | | - | | 1:2000 | |
| Anti-NDUFS3 | | Abcam (ab183733) | | rb | | - | | 1:2000 | |
| Anti-NDUFB10 | | Abcam (ab196019) | | rb | | - | | 1:5000 | |
| Anti-COX7A2L [SCAFI] | | St Johns Laboratory (STJ110597) | | rb | | - | | 1:1000 | |
| Anti-SDHA | | Abcam (Ab14715) | | ms | | - | | 1:2000 | |
| Anti-SYP | | Santa Cruz Biotechnology (sc-17750) | | ms | | - | | 1:2000 | |
| Anti-TH (H-196) | | Santa Cruz Biotechnology (sc-14007) | | rb | | 1:200 | | - | |
| Anti-GHITM [TMBIM5] | | Proteintech (16296-1-AP) | | rb | | - | | 1:1000 | |
| Anti-TUJ-1 | | GRP (GT11710) | | ms | | 1:400 | | 1:2000 | |
| Anti-VDAC 1/2 | | Proteintech (10866-1-AP) | | rb | | - | | 1:2000 | |
| Anti-Acetylated Tubulin | | Sigma (T7451) | | ms | | 1:4000 | | - | |
|  | |  | |  | |  | |  | |

Table 3: List of used secondary antibodies.

**Secondary Antibody Manufacturer Source Dilution**

**For Immunofluorescence (IF):**

Alexa Fluor 488 Anti-mouse Invitrogen (A21131) Goat 1:800

Alexa Fluor 555 Anti-mouse Invitrogen (A21147) Goat 1:800

Alexa Fluor 488 Anti-rabbit abcam (ab150077) Goat 1:800

Alexa Fluor 555 Anti-rabbit abcam (ab150078) Goat 1:800

**For Immunoblotting (IB):**

Goat Anti-Mouse IgG H&L Biozol (LIN-ITC207P) Goat 1:2000

Goat Anti-Rabbit IgG H&L Biozol (LIN-ITC208P) Goat 1:2000

Table 4: List of dyes used

**Dye Manufacturer (Cat. No)**

BioTracker ATP-Red Live cell dye Sigma-Aldrich (SCT045)

Fura-2 AM Thermo Fisher (F1221)

FxCycle PI/RNAse Solution Thermo Scientific (F10797)

Mito Tracker Deep Red FM (MTDR) Thermo Fisher (M22426)

Mito Tracker Green FM (MTG) Thermo Fisher (M7514)

Mitochondrial Superoxide Indicator (MitoSox) Thermo Fisher (M36008)

Table 5: List of used kits.

**Name Manufacturer**

Monarch Total RNA Miniprep Kit New England BioLabs (T2010S)

Seahorse XF Cell Mito Stress Test Kit Agilent (103010-100)

RevertAid First Strand cDNA Synthesis Kit Thermo Scientific (K1621)

SuperSignal West Pico PLUS Chemilum. Substrate Thermo Scientific (S8761)

TurboFectin 8.0 OriGene (TF81001)

# Supplementary Protocols

## Differentiation and maturation of NPC to neurons

The differentiation and maturation of NPC to neurons was conducted following usual protocols.

The base medium N2B27 for NPC was composed of DMEM/F-12 (Thermo Fisher, #11320) and Neurobasal Medium (Thermo Fisher, #) at a 1:1 ratio, supplemented with 1:200 diluted N2 supplement (Thermo Fisher), 1:100 diluted B27 supplement without vitamin A (Thermo Fisher) and 1:100 diluted penicillin/streptomycin/glutamine (PSG, Thermo Fisher). Culture medium was exchanged daily and cells were split every 5–7 days when confluency of at least 80% was reached. At high cell numbers, the volume of cell culture medium was increased. Splitting was performed at a ratio of 1:12–1:20 with accutase (Sigma-Aldrich) treatment for approximately 5 min at 37°C to generate a single-cell suspension. Earlier passages were expanded in a lower splitting ratio of 1:8. Next, the NPC suspension was transferred to a falcon containing DMEM/F-12 with 0.1% bovine serum albumin (BSA, Thermo Fisher) to stop the enzymatic digestion and centrifuged at 300 g for 4 min. Finally, the cell pellet was resuspended in NKM and seeded out in fresh Matrigel-coated 6-well plates.

A day prior to the start of the differentiation, 300000 NPCs were plated onto a Poly-L-Ornithine (PLO)-coated well of a 12-well plate in NKM. The medium was changed after 24 h to Neuronal Induction Medium (NIM) to initiate the differentiation process of the NPC. Therefore, NIM contains 1 µM SAG, 75 µM Ascorbic Acid, and the neurotrophic growth factors BDNF and GDNF added each 2 ng/ml in addition to the base medium N2B27. This medium was exchanged daily for five days. On day 6 to day 9 Neuronal Differentiation Medium^+^ (NDM^+^) was used and medium change was performed every second day. On day 10 the Activin A was removed from the medium so that the cells were maintained in Neuronal Differentiation Medium (NDM) until the end of the differentiation on day 23. A schematic representation of the differentiation process from NPC to neurons is shown in Figure S 2A. Cells were carefully re-plated with Accutase between day 8 and 12 depending on the experiment. An additional re-plating of neurons was performed by washing the cells with medium instead of PBS and carefully detaching them with TripLE (Gibco). After long-term culture on PLO the neuronal networks can detach from the well. Thus, NDM was aspirated with a manual pipette instead of vacuum when performing media changes.

Maturation was controlled by the gene expression profile of markers for pluripotency respectively neurons in culture samples taken in intervals of 5-6 days. The mRNA expression of the pluripotency markers SRY (sex determining region Y)-box 2 (Sox-2), octamer-binding transcription factor 4 (Oct-4) and Krüppel-like Factor 2 (KFL-2) were significantly decreased in samples from day 23 (neurons) compared to day 0 (NPC) of differentiation (Figure S 2B). Furthermore, the genes encoding for the Microtubule-associated protein 2 (MAP2) and the Growth Associated Protein 43 (GAP43) showed an increasing trend for mRNA expression throughout the differentiation process, resulting in a significant upregulation of these genes in neurons compared to NPC. An immunofluorescence co-staining of MAP2 and the neuron-specific class III beta-tubulin (TUJ-1) showed numerous neurons in differentiated cell culture (Figure S 2C), which goes in line with the increase of MAP2 gene expression. Additionally, a 1:4000 IF staining of acetylated Tubulin indicated specification of TUJ-1+ cells into axonal sub-compartments of neurons (Figure S 2D), which are required for functional neurons. To verify the functionality of the cell model, differentiated neurons were electro-physiologically characterized by multi-electrode array (MEA) measurements. To identify neurotransmitter responsive network activity, different pharmacological agonistic and antagonistic modulators were applied as shown in Figure S 2G. The neuronal activity determined by these measurements revealed a mixed neuronal culture, which was primary responsive to glutamate/glycine and dopamine. The presence of neuronal subtypes in the culture were shown by immunofluorescence co-staining of neurons with Tyrosine hydroxylase (TH) and choline-acetyl transferase (CHAT). Tyrosine hydroxylase is an important enzyme catalyzing L-DOPA production, which is an intermediate required for dopamine synthesis. Figure S 2E shows a subpopulation of TH+ neurons, indicating the presence of dopaminergic neurons in the culture. Staining of CHAT revealed neurons, that contain the transferase enzymes for the synthesis of the neurotransmitter Acetyl Choline, indicating the presence of cholinergic neurons.

# Supplementary Figures

**Figure S 1: Mdivi-1 affects cell growth and mitochondrial biogenesis.** (A) Growth curves of HeLa cells treated with Mdivi-1 for 24h, respectively 1 w, before recording. Left: pre-treatment with Mdivi-1, no Mdivi-1 during recording; right: ongoing treatment with Mdivi-1 (right). (B) Expression of p21 is elevated in Mdivi-1 treated HeLa cells (N=4, n=16, ANOVA. (C) Midivi-1 effects on cellular morphology of HeLa cells stained with Hoechst (scale bar: 1000 µm). (D) Circularity of HeLa cells with acute and long-term Mdivi-1 and Rotenone treatment (N=4 independent experiments, n=13, ANOVA). (E) Exemplary cLSM images and MiNA skeleton of control and Mdivi-1-treated HeLa cells stained with Mito Tracker™Green (MTG) (scale bar: 10 µm). (F) Treatment with Mdivi-1 (50 µM) leads to increased, while Rotenone (1 µM) leads to decreased mitochondrial mass (N=6; nDMSO=828, nMdivi-1 10 µM 24h=519, nMdivi-1 50 µM 24h=522, nDrp1 K38A mt EGFP=28, nRotenone=196; DMSO median as dashed line). (G) Accute Mdivi-1 treatment indicates elevation in gene expression of TFAM in HeLa cells (N=4, n=16). (H) Protein levels of Voltage Dependent Anion channel (VDAC) normalized on *β*-III-Tubulin (TUJ-1) are increased in Mdivi-1-treated cells (left panel: HeLa n=3, NPC, n=2 right panel: neurons n=11, all ANOVA).

Figure S 2: NPC differentiation into neurons. (A) Schematic overview of NPC differentiation process with exemplary cell culture images. (B) Relative gene expression of markers for pluripotency (Sox-2 and Oct-4) decrease, while markers for neurons (MAP2 and GAP43) increase during the differentiation process (N=1, n=9). (C, D, E) immune staining of NPC-derived neurons for neuronal markers. (F) Exemplary image of neurons post transferring on a multi electrode array chip. (G) Electrophysiological characterization of neuronal culture (N=3, n=9).

**Figure S 3: Effects of Mdivi-1 on mitochondrial respiration in neurons**. (A) Mito Stress test to determine OCR in neurons, inhibitors for ATP synthase (oligomycin¸ 1 μM), uncoupler trifluoromethoxy carbonyl cyanide phenylhydrazone (FCCP, 1 μM) and inhibitors for complex I (Rot., rotenone; 0.75 μM) and complex III (AA; antimycin A; 1 μM) were subsequently added. Oxygen consumption rates (OCR) were determined with an automatic flux analyzer (Seahorse XF96/Agilent) and normalized to cell numbers (left panel) or mitochondrial mass (right panel). The normalization on the mitochondrial mass was done by using the factor that describes the mean protein level of VDAC normalized to TUJ-1. (B, C) Mitochondrial basal, ATP production-related and maximal respiration, non-mitochondrial oxygen consumption and proton leak in living cells (control and Mdivi-1 treated) were determined by OCR [pmol/min/1000cells] or containing additional normalization on the mitochondrial mass. (N=1, n>=8, left panel all tested with KW, right panel statistic test indicated). (D) Determination of N-respirasome, Q-respirasome and CIV complex activities via Seahorse in neurons (N=1, n=8). KW: Kruskal-Wallis, ANOVA test where indicated.

**Figure S 4**: **Mdivi-1 interferes with CI-dependent SC assembly**. (A) Blue Native gel with isolated mitochondria of Mdivi-1 treated HeLa cells and immunoblotting of NDUFB10 shows an increased intermediate CI assembly form.

**Figure S 5: Supplementary Results for molecular docking.** (A) Alignment of structures of murine and bovine CI. (B) Quantification of identical interactions between bovine CI rotenone, Q1, Q10 and Mdivi-1 shows less interactions compared to the redocked piercidin A in murine CI (left=comparison of individual best poses generated by Autodock, right=pooled poses of all ligands, n=5 poses per structure). (C) Respective binding energy of best docking poses (n=5 per condition) and parameters of pooled docking poses (N=4 docking processes, n=25 docking poses).

**Figure S 6: Neuronal Ca^2+^-response is reduced in Mdivi-1 treated cells. (**A) Representative Fura-2 traces of DMSO treated neurons after stimulation with glutamate/glycine (100 µM each). (B) Calcium imaging before (N=2, n=5, n=4, n=4, ANOVA) and after stimulation (N=2, n=6, n=4, n=1, ANOVA).


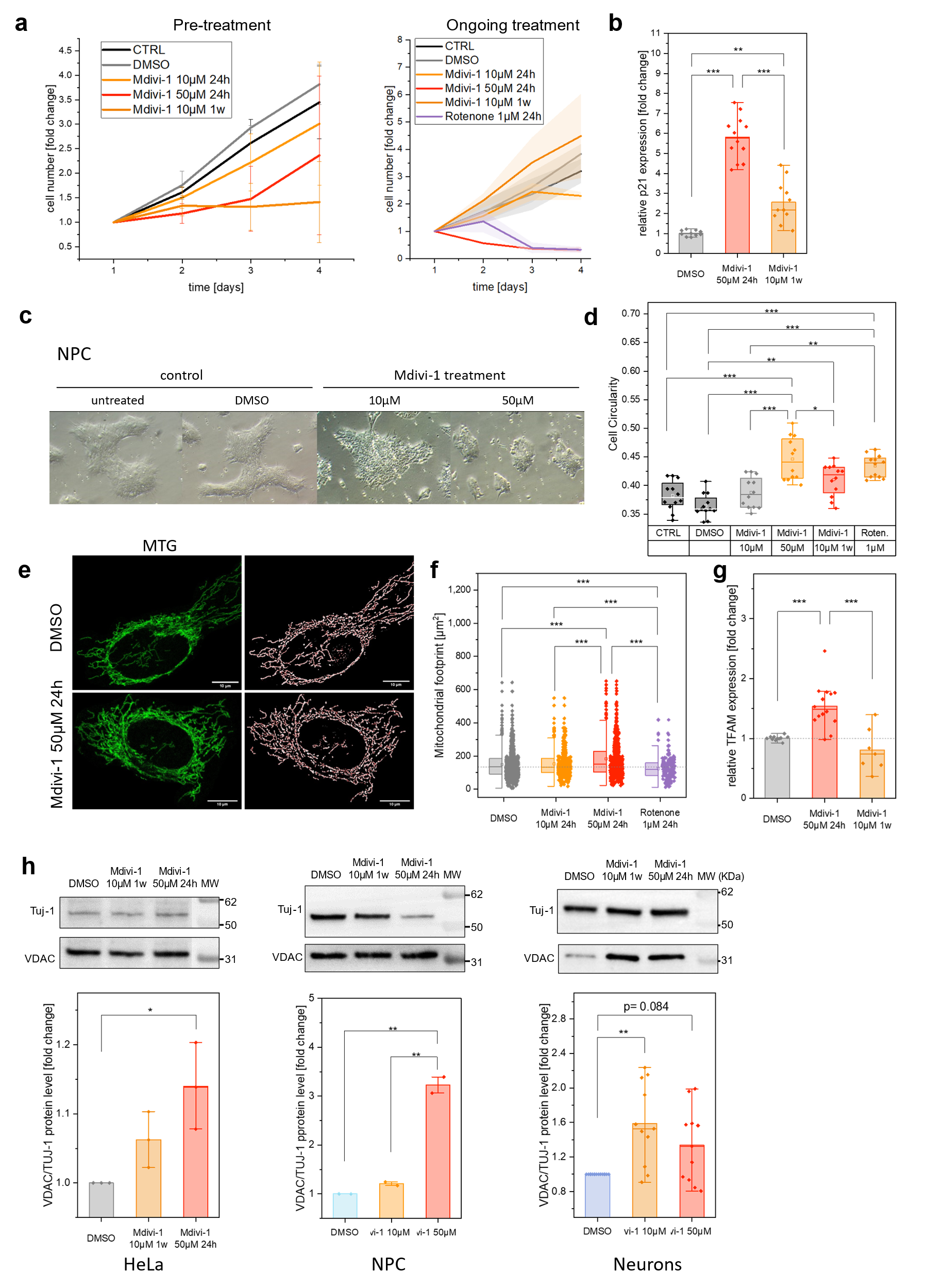


Figure S 1


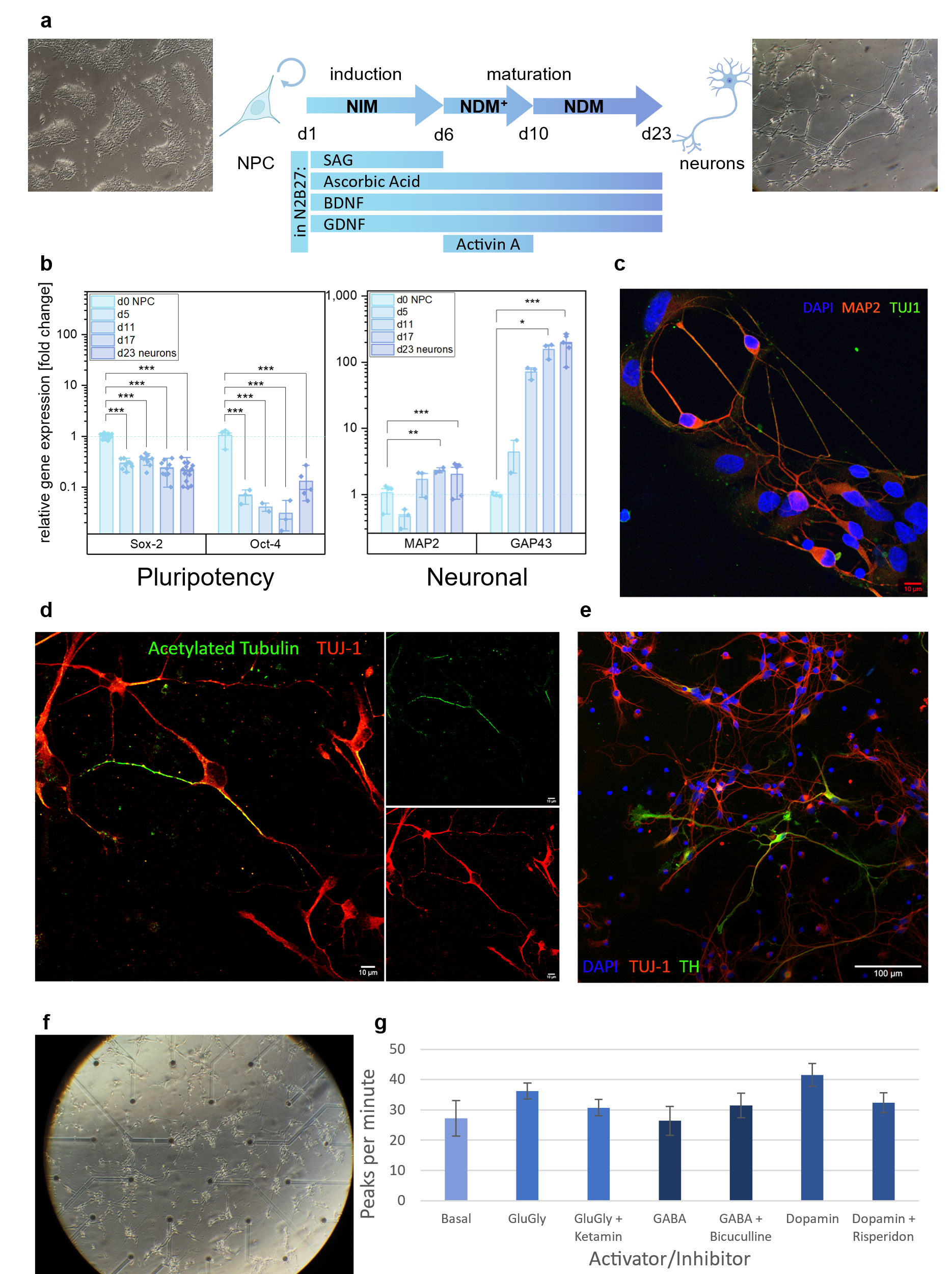


Figure S 2

**
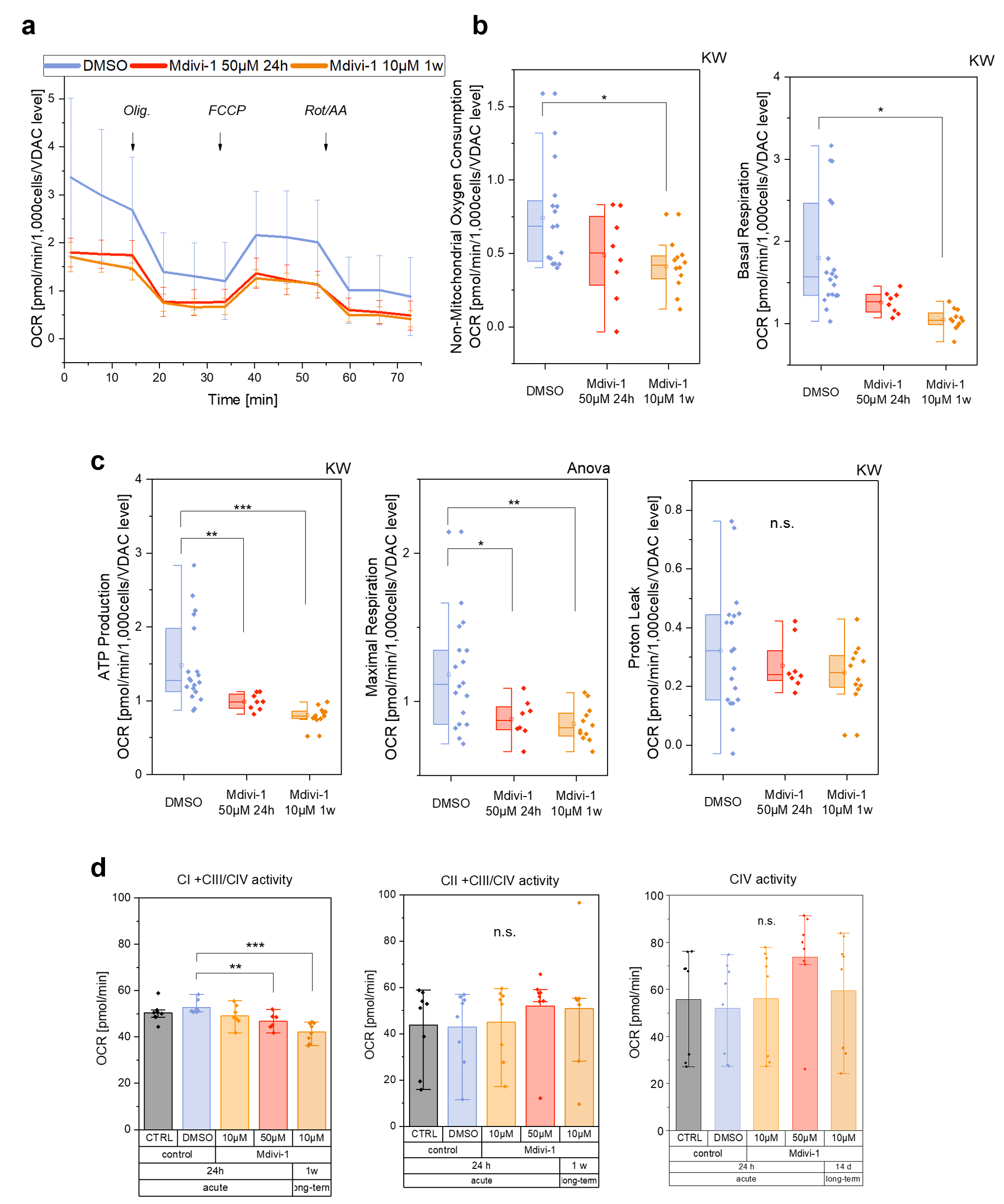
**

Figure S 3


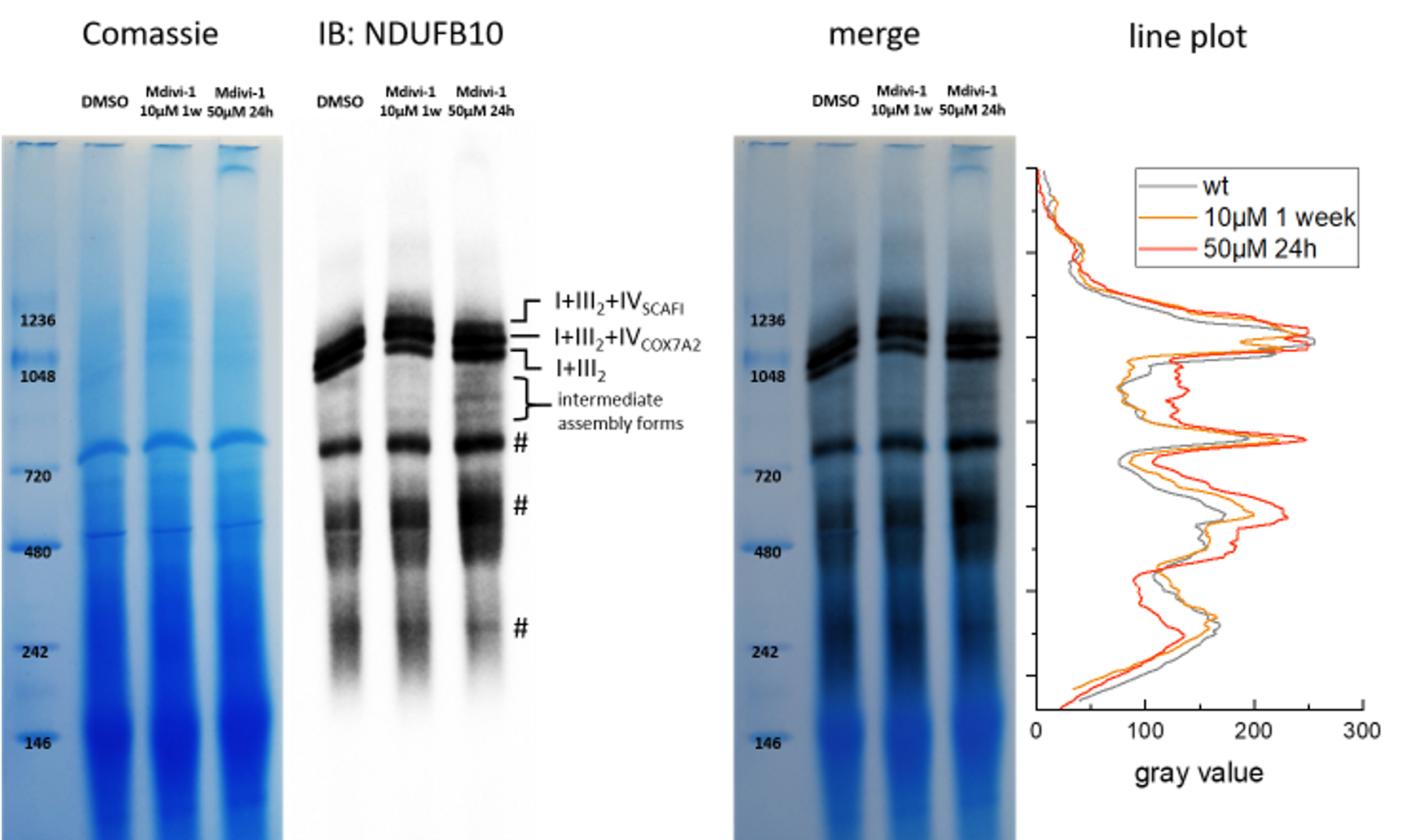


Figure S 4

**
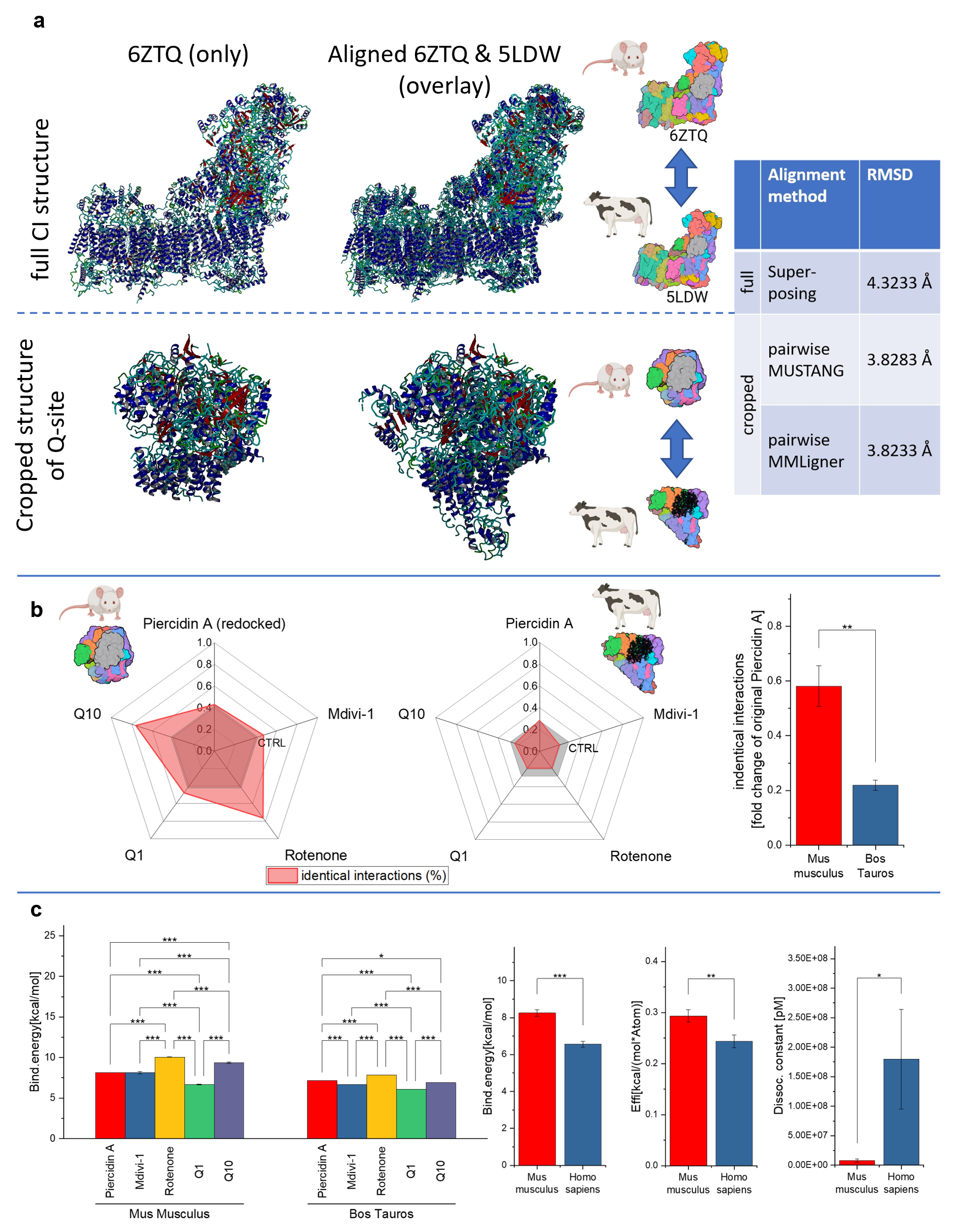
**

Figure S 5


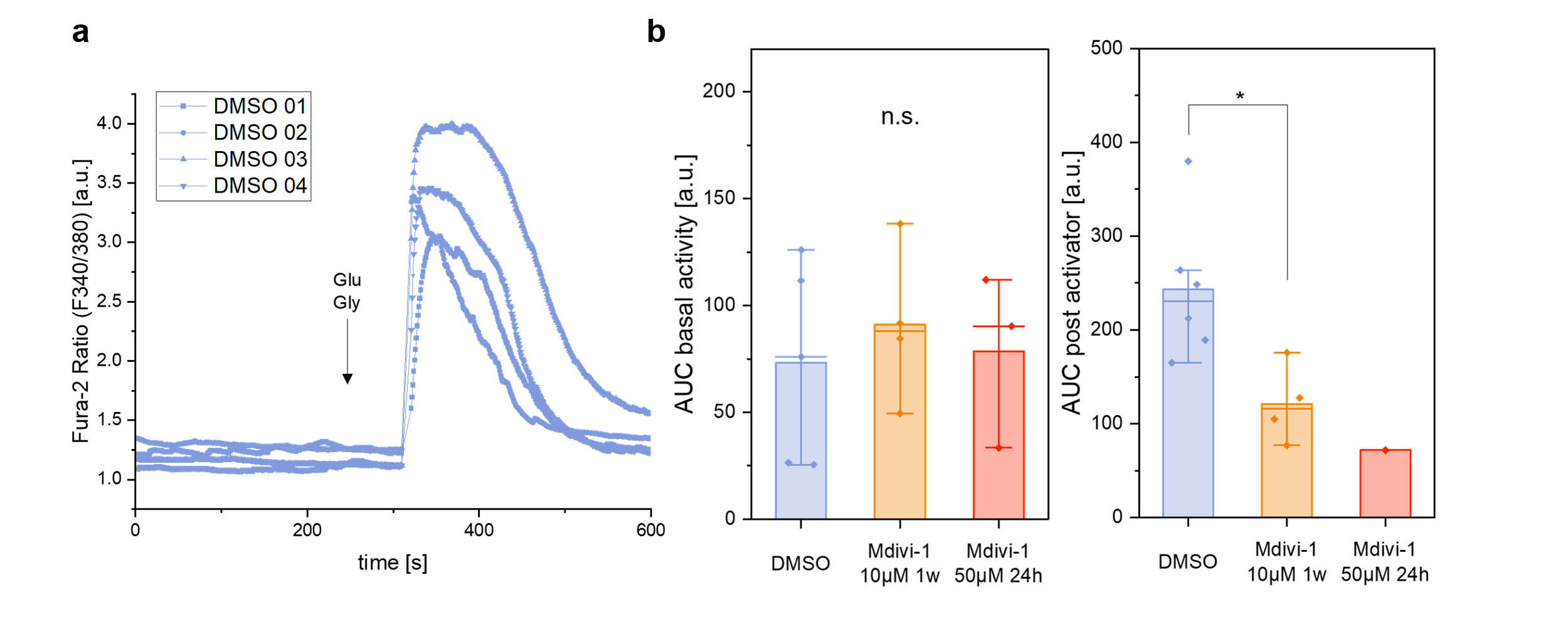


Figure S 6
