# Supplementary figures and images for: Detailed analysis of Mdivi-1 effects on complex I and respiratory supercomplex assembly
Source: Sci Rep. 2024 Aug 24;14:19673. doi: 10.1038/s41598-024-69748-y (PMC11347648; doi:10.1038/s41598-024-69748-y)

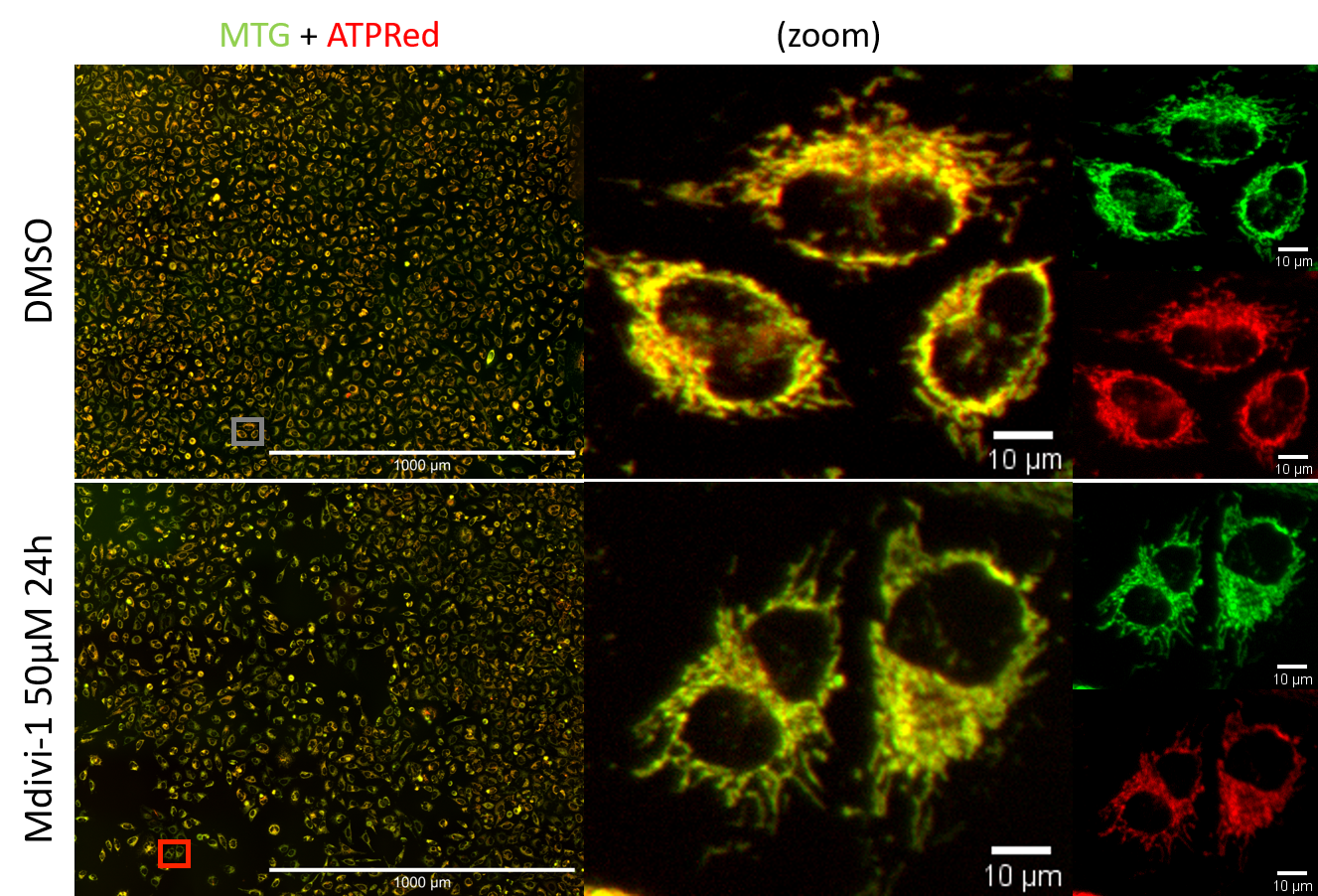

Supplement: Supplementary file 4 — Supplementary Information 4. [file 41598_2024_69748_MOESM4_ESM.png]

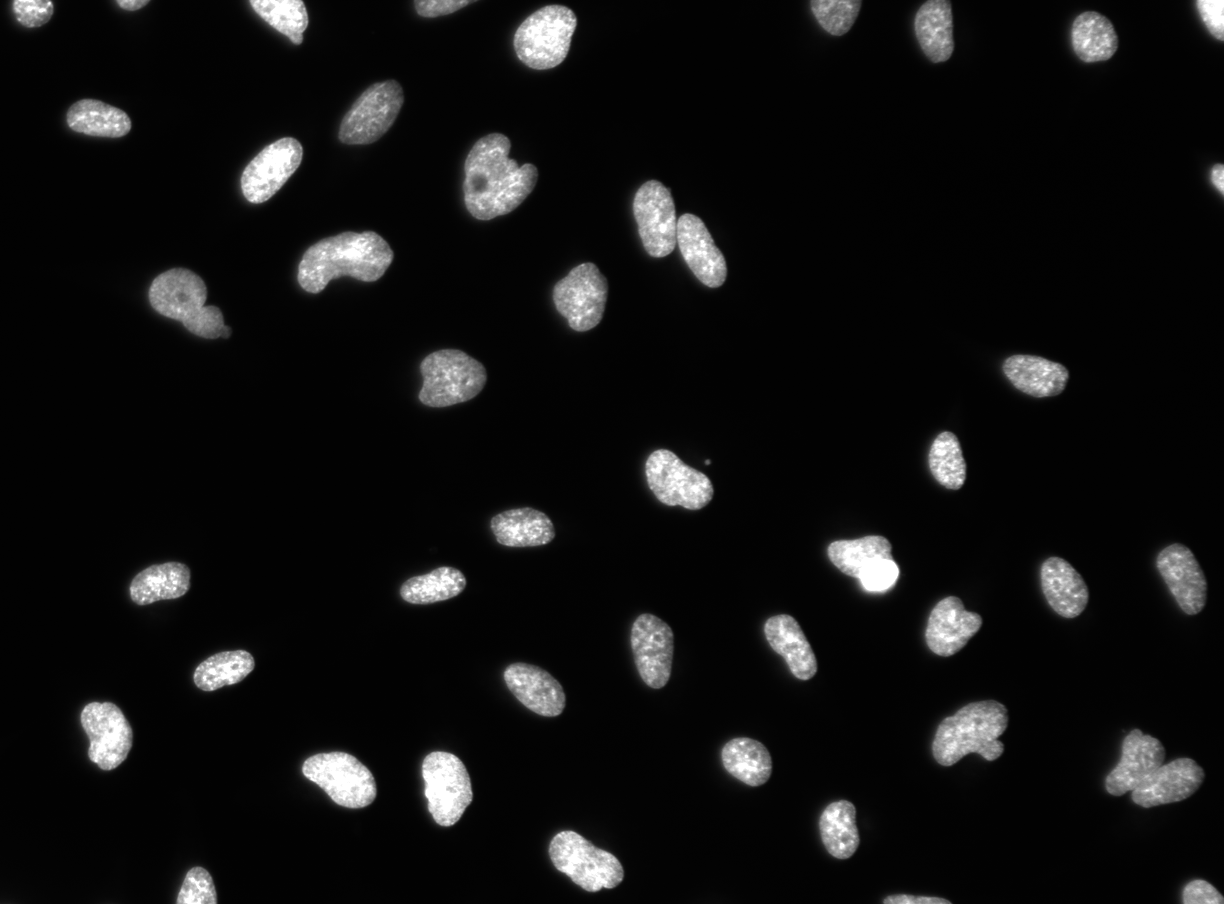

Supplement: Supplementary file 5 — Supplementary Information 5. [file 41598_2024_69748_MOESM5_ESM.tif]

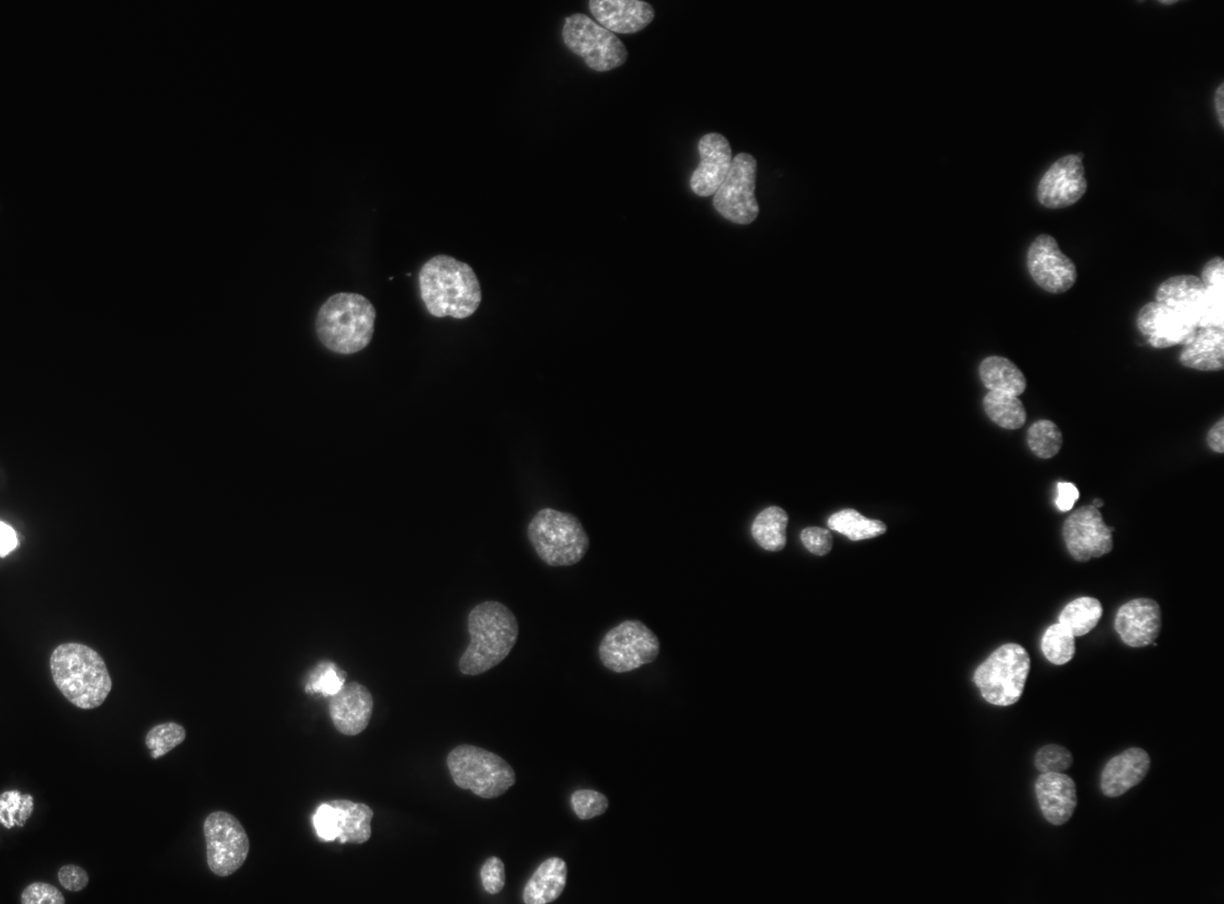

Supplement: Supplementary file 6 — Supplementary Information 6. [file 41598_2024_69748_MOESM6_ESM.tif]

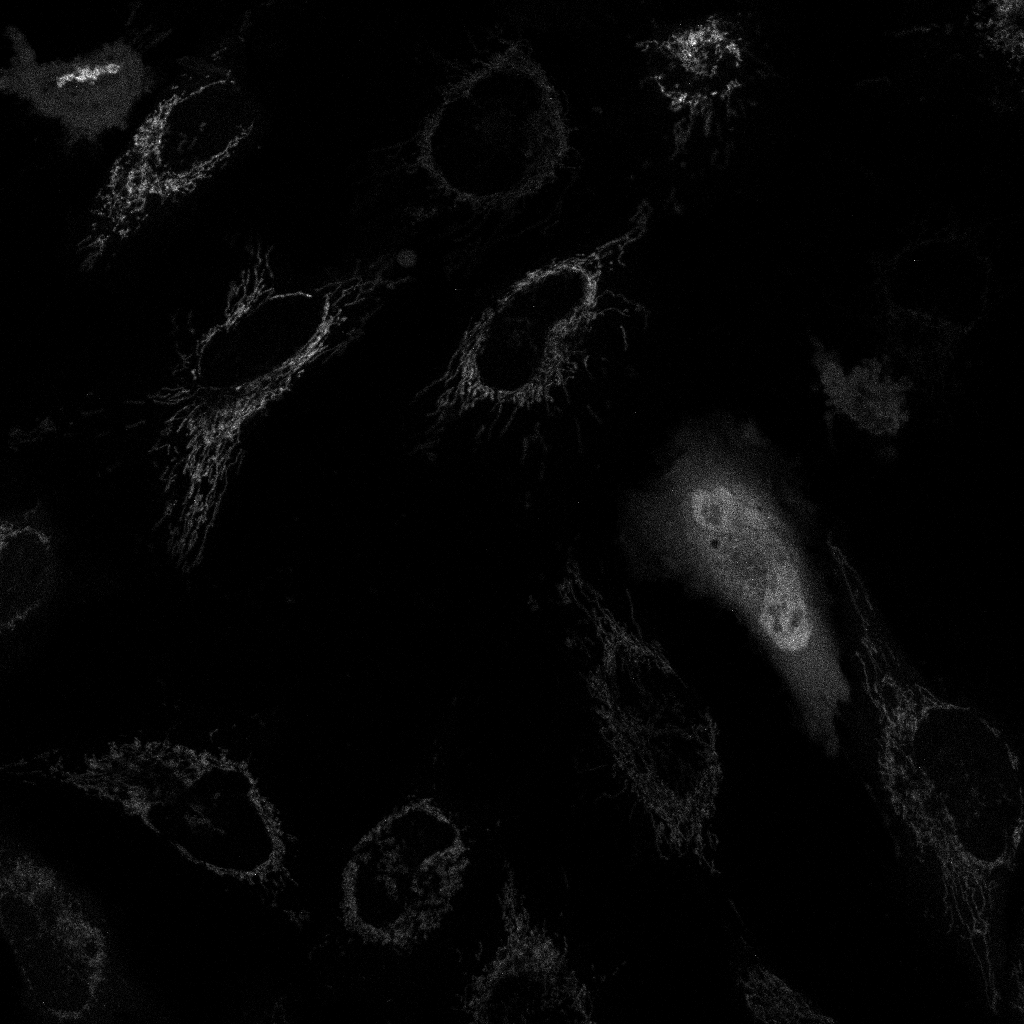

Supplement: Supplementary file 8 — Supplementary Information 8. [file 41598_2024_69748_MOESM8_ESM.tif]

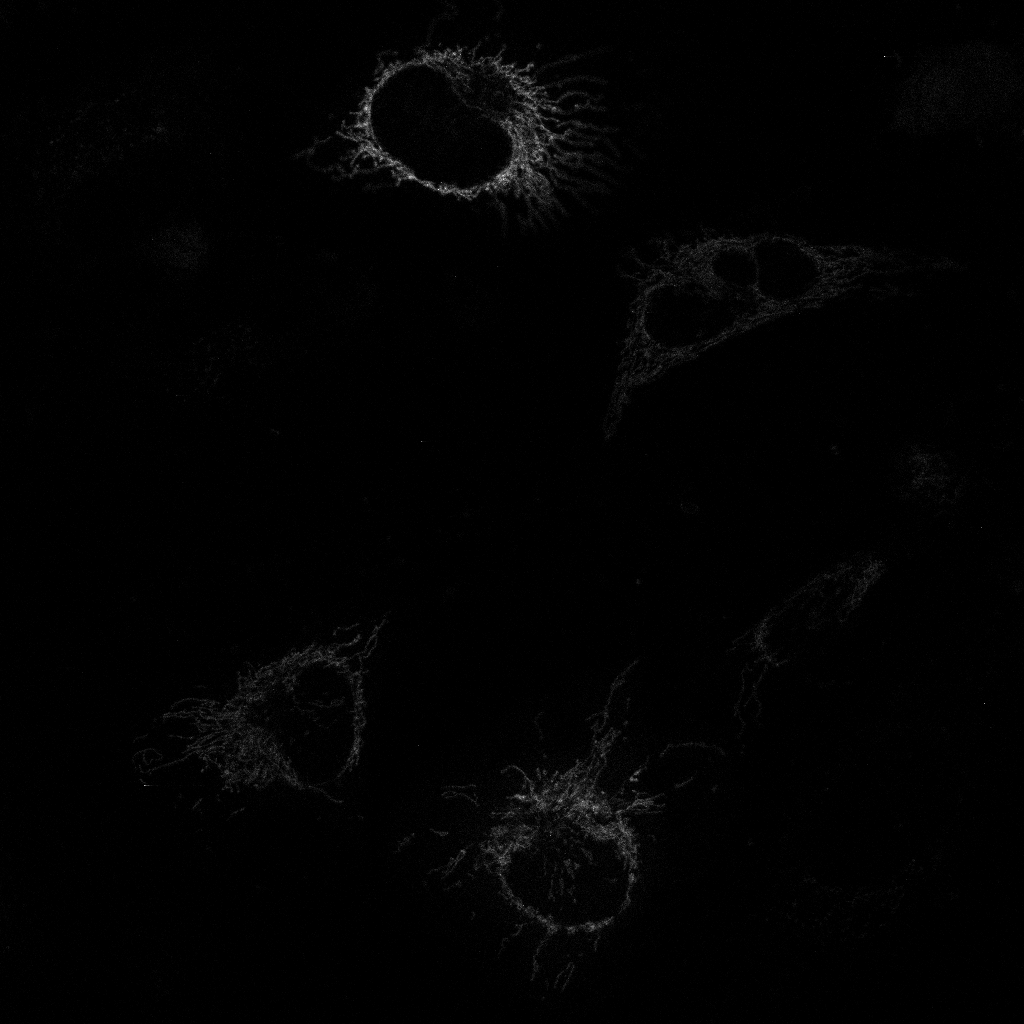

Supplement: Supplementary file 9 — Supplementary Information 9. [file 41598_2024_69748_MOESM9_ESM.tif]

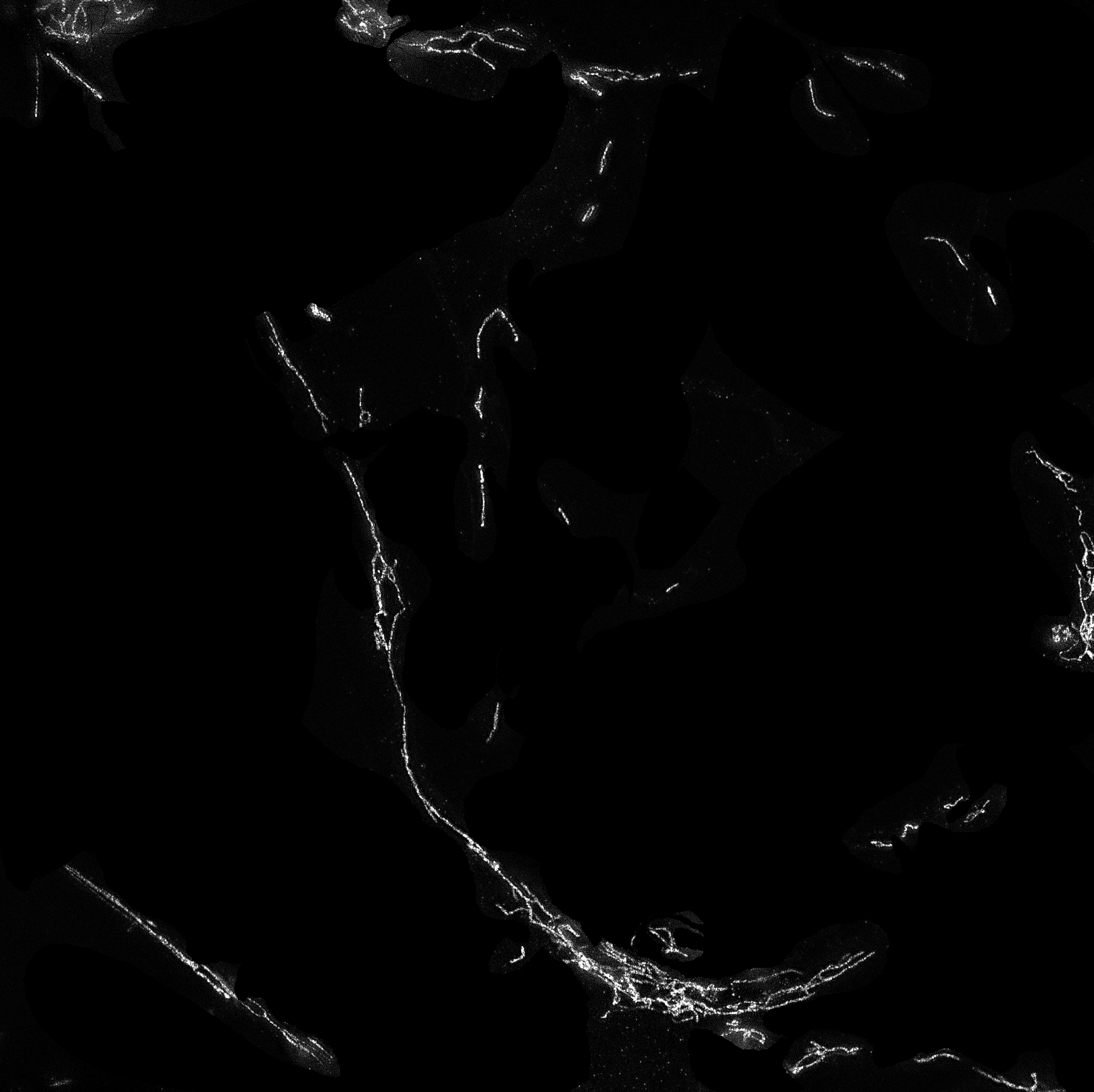

Supplement: Supplementary file 12 — Supplementary Information 12. [file 41598_2024_69748_MOESM12_ESM.tif]

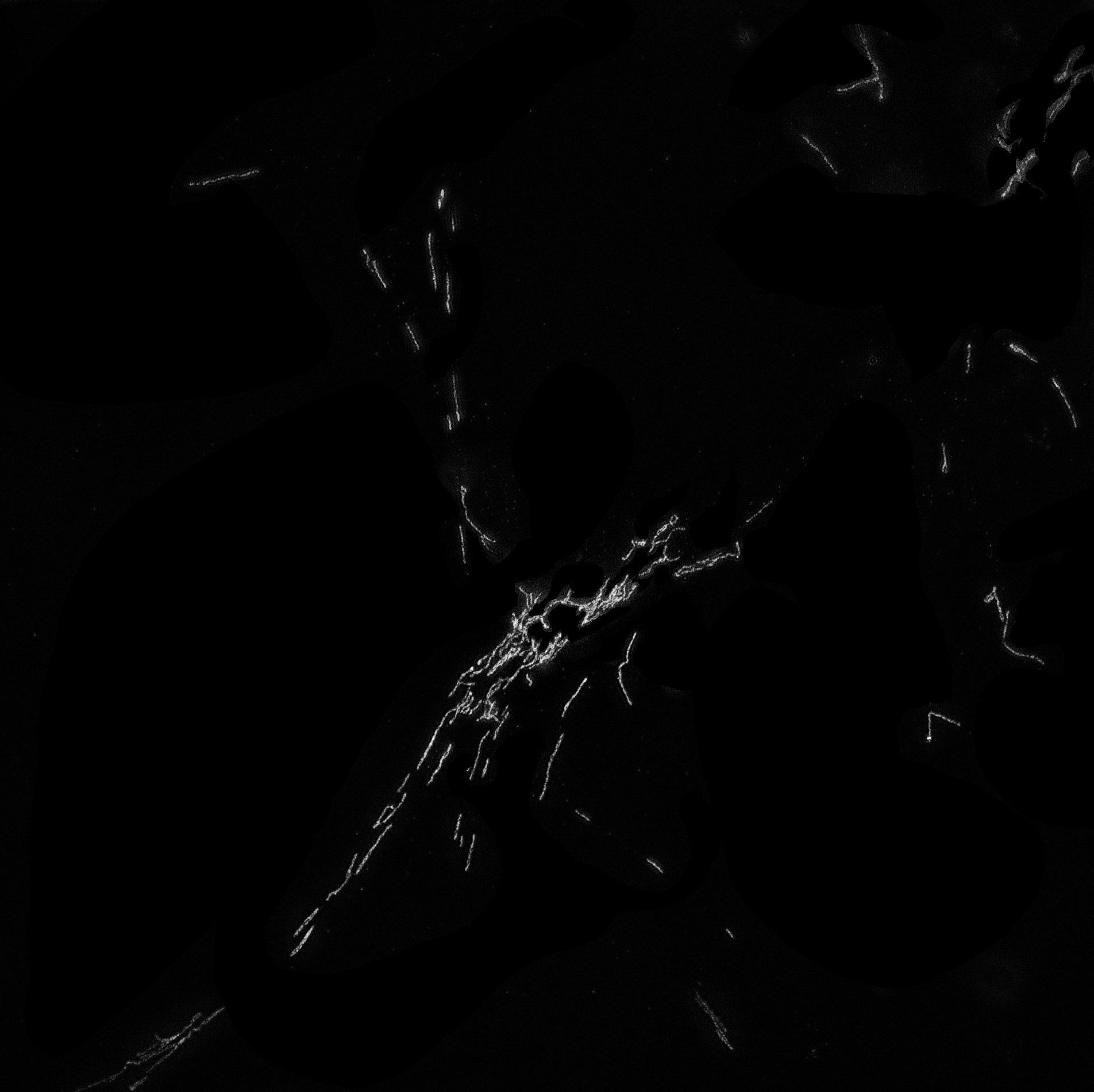

Supplement: Supplementary file 13 — Supplementary Information 13. [file 41598_2024_69748_MOESM13_ESM.tif]
