# Supplementary material for: Detailed analysis of Mdivi-1 effects on complex I and respiratory supercomplex assembly
Source: Sci Rep. 2024 Aug 24;14:19673. doi: 10.1038/s41598-024-69748-y (PMC11347648; doi:10.1038/s41598-024-69748-y)
Supplement: Supplementary file 14 — Supplementary Information 14. [file 41598_2024_69748_MOESM14_ESM.pdf]

SCAFI & NDUFB10 blots

1 DMSO
2 Mdivi-1 10µM 1w
3 Mdivi-1 50µM 24h

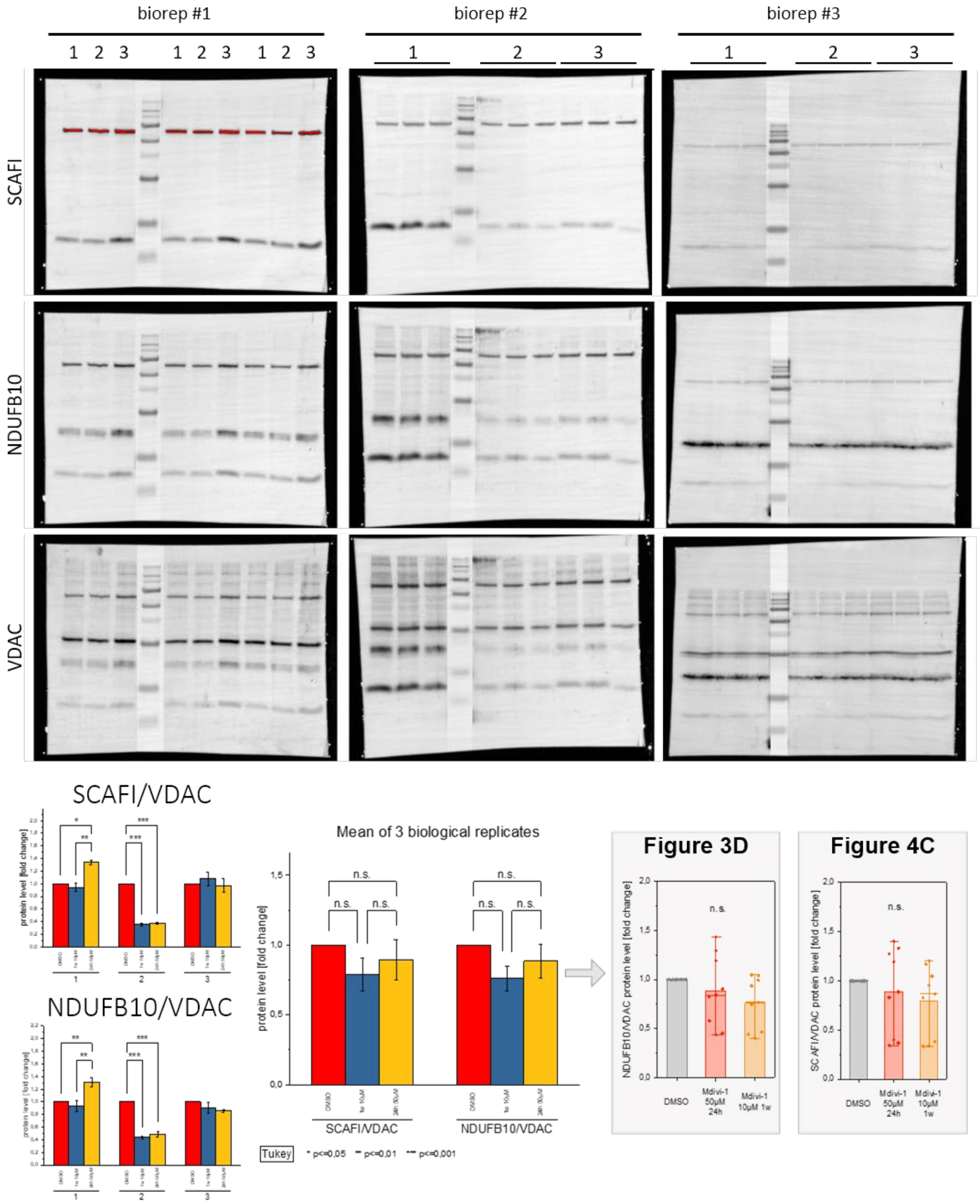

Source data SCAFI-NDUFB10

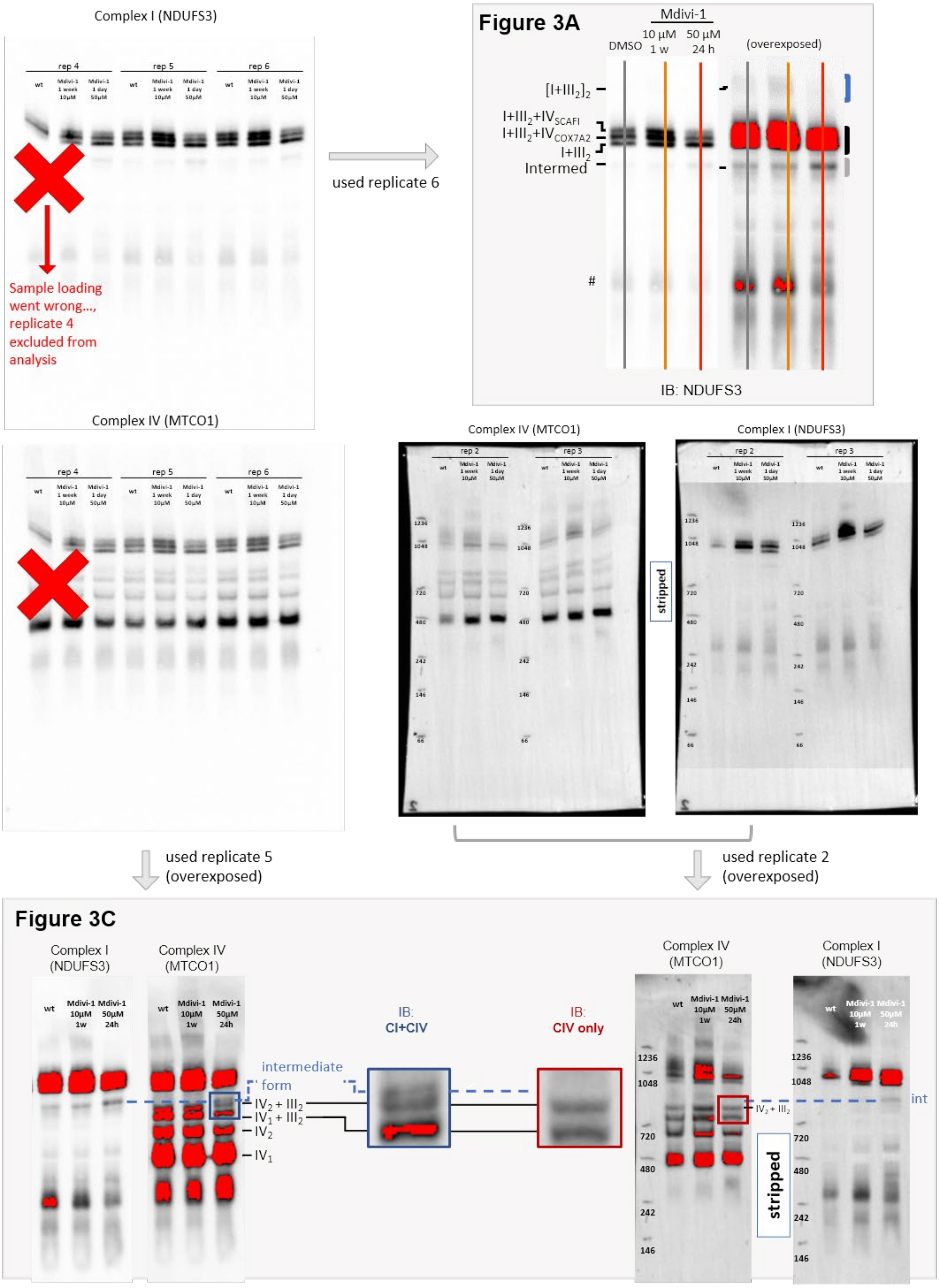

Source data Figure 3

Complex IV (MTCO1)

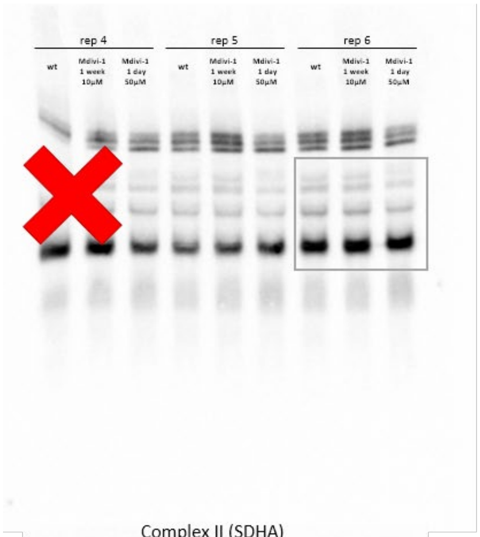

used replicate 6

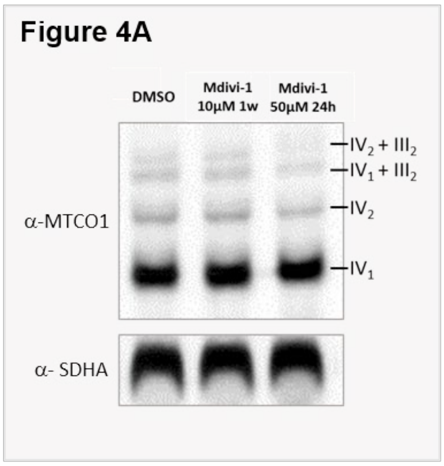

Complex II (SDHA)

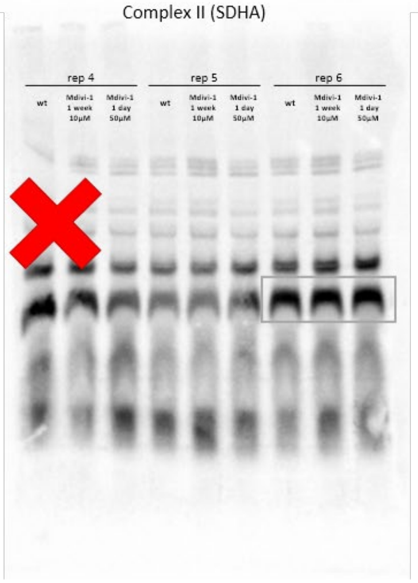

Source data Figure 4

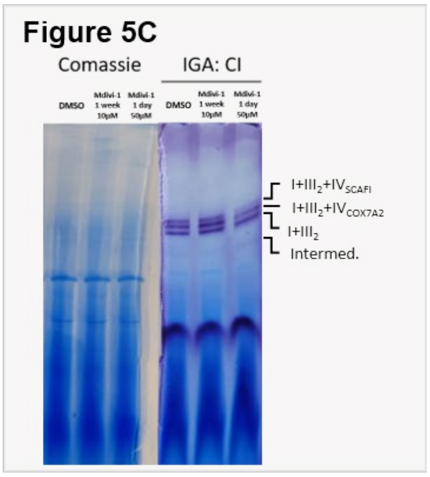

Comassie

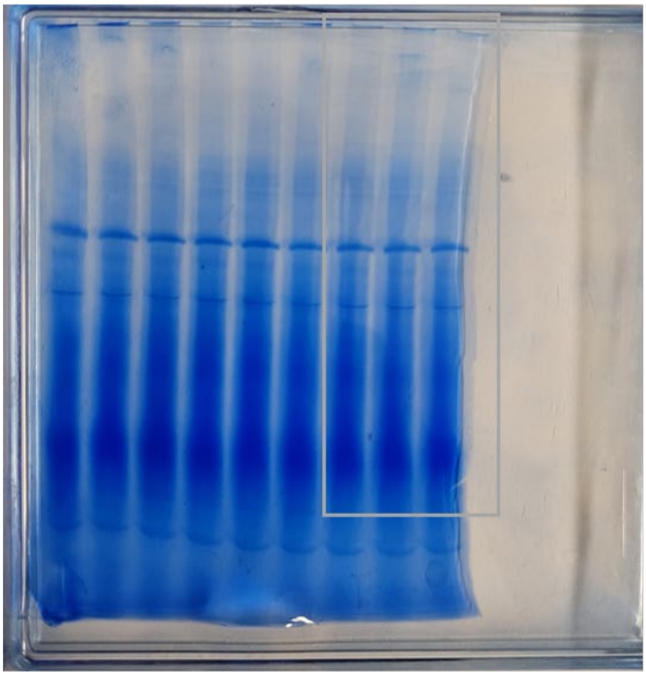

IGA: CI
(1h)

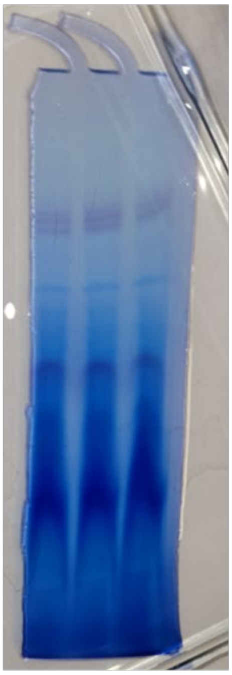

IGA: CI
(24h)

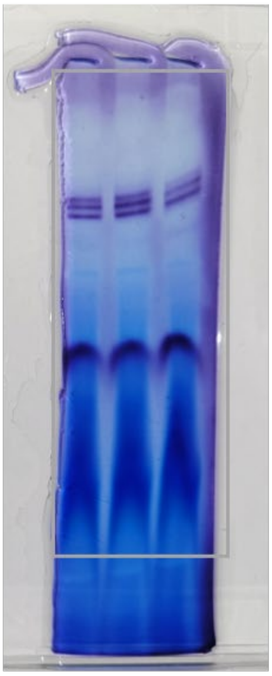

Source data Figure 5

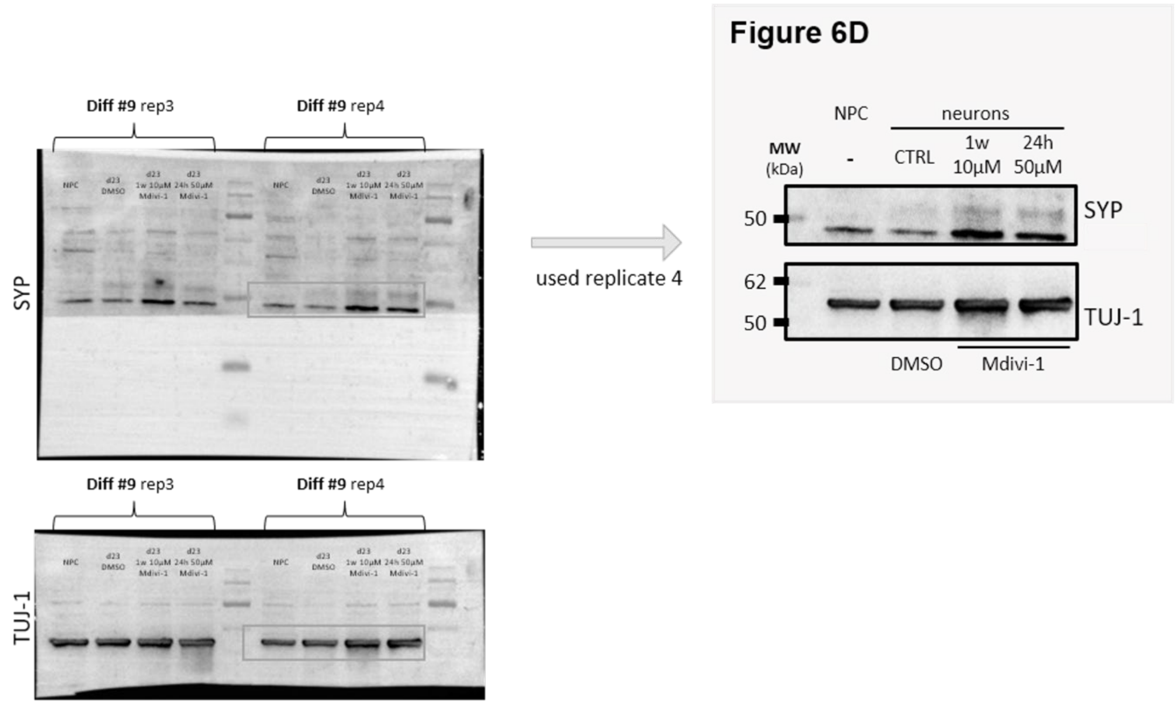

Source data Figure 6

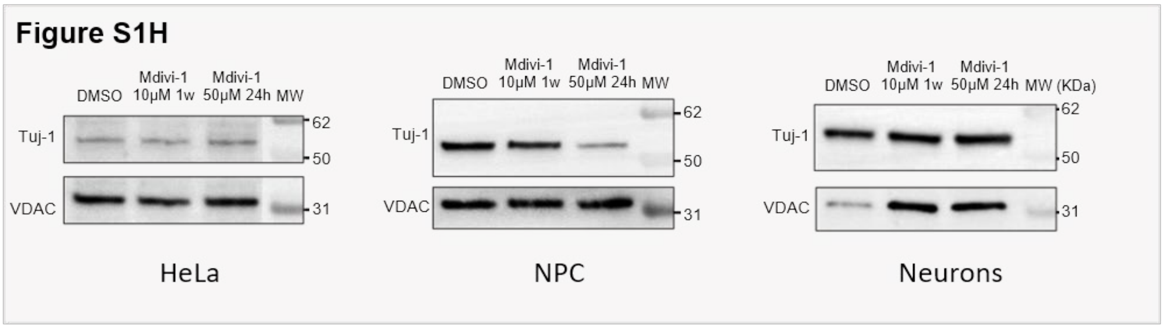

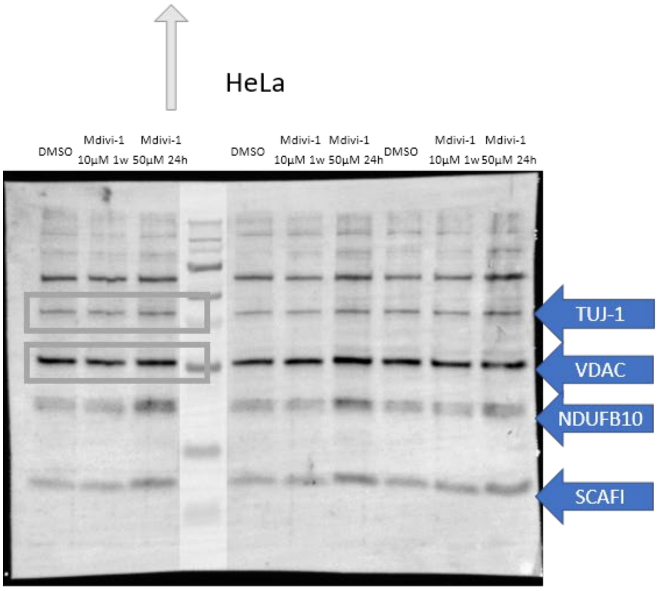

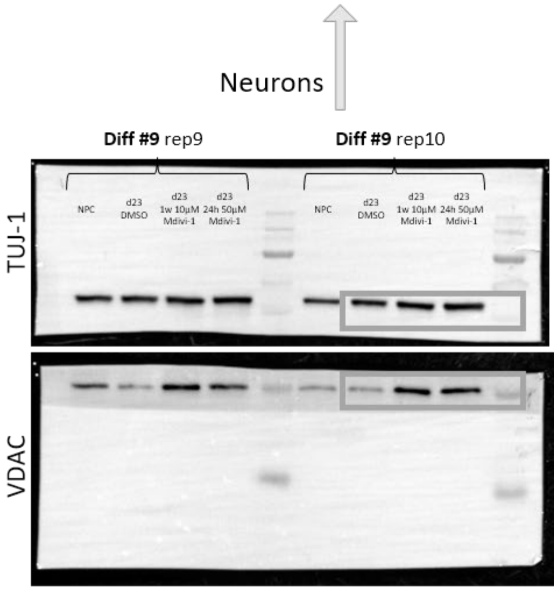

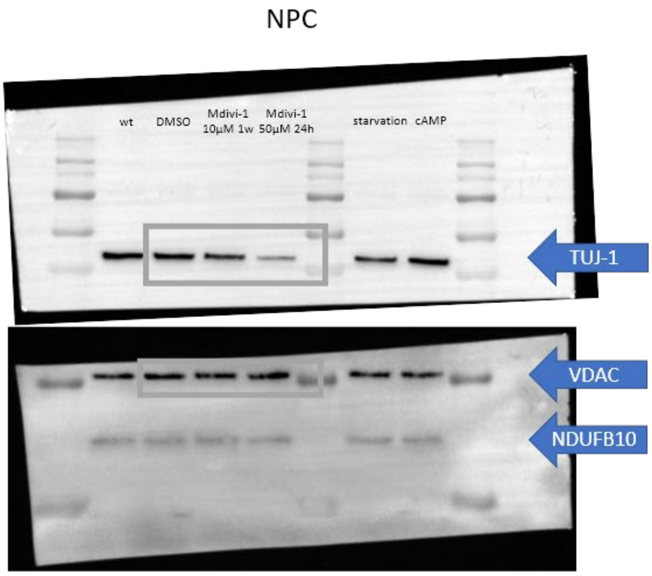

Source data Figure S1

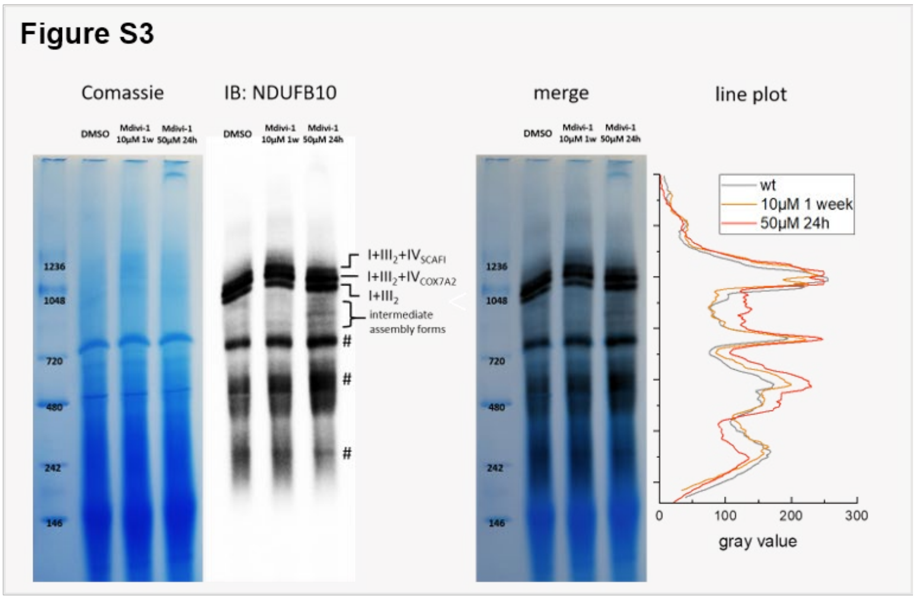

Comassie

IB: NDUFB10

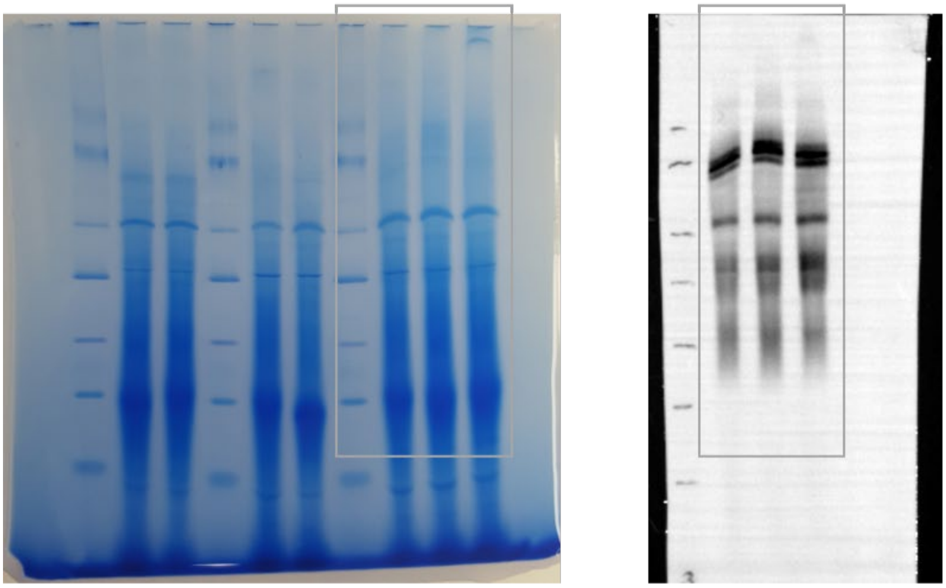

Source data Figure S3
